# A Plug-and-Play Human-Centered Virtual TEDS Architecture for the Web of Things

**DOI:** 10.3390/s18072052

**Published:** 2018-06-27

**Authors:** Dixys L. Hernández-Rojas, Tiago M. Fernández-Caramés, Paula Fraga-Lamas, Carlos J. Escudero

**Affiliations:** 1 Department of Computer Science, Academic Unit of Civil Engineering, Universidad Técnica de Machala, 070150 Machala, Ecuador; 2 Department of Computer Engineering, Faculty of Computer Science, Universidade da Coruña, 15071 A Coruña, Spain; tiago.fernandez@udc.es (T.M.F.-C.); paula.fraga@udc.es (P.F.-L.); escudero@udc.es (C.J.E.)

**Keywords:** sensors, actuators, TEDS, IEEE 21451, Internet of Things, Web of Things, smart sensors, smart transducers, interoperability, human-centered services

## Abstract

This article presents a Virtual Transducer Electronic Data Sheet (VTEDS)-based framework for the development of intelligent sensor nodes with plug-and-play capabilities in order to contribute to the evolution of the Internet of Things (IoT) toward the Web of Things (WoT). It makes use of new lightweight protocols that allow sensors to self-describe, auto-calibrate, and auto-register. Such protocols enable the development of novel IoT solutions while guaranteeing low latency, low power consumption, and the required Quality of Service (QoS). Thanks to the developed human-centered tools, it is possible to configure and modify dynamically IoT device firmware, managing the active transducers and their communication protocols in an easy and intuitive way, without requiring any prior programming knowledge. In order to evaluate the performance of the system, it was tested when using Bluetooth Low Energy (BLE) and Ethernet-based smart sensors in different scenarios. Specifically, user experience was quantified empirically (i.e., how fast the system shows collected data to a user was measured). The obtained results show that the proposed VTED architecture is very fast, with some smart sensors (located in Europe) able to self-register and self-configure in a remote cloud (in South America) in less than 3 s and to display data to remote users in less than 2 s.

## 1. Introduction

The Internet of Things (IoT) is no longer a novel concept and has already become an everyday term. In fact, the IoT market has maintained a sustainable growth in the last several decades and is estimated to grow to 20 billion devices in 2020 [1]. IoT infrastructure engages in multiple application domains and in emerging businesses where physical and virtual devices are able to inter-operate through a data network without human intervention [2]. Examples of IoT applications can be found for smart cities [3], smart homes [4,5], e-health [6], intelligent transportation [7,8,9], critical infrastructures [10], precision agriculture [11], smart factories [12,13,14], or for Industry 4.0 [15,16,17,18]. These applications are increasingly heterogeneous, complex, and demanding [19].

At the foundations of every IoT platform are the “things” that are intelligent sensors and actuators usually connected to wireless networks (thus creating a Wireless Sensor Network (WSN)). The information of such elements can be managed locally or from higher layers (through a gateway, a cloud computing system, and even through a blockchain [20]).

Due to the need for a greater abstraction level to implement advanced applications, the IoT has had to evolve. Thus, intelligent sensors, besides being able to process the collected information, have to perform additional computing tasks, exchange data through wired or wireless networks, add connectivity with actuators, or include some kind of display [21]. In addition, sensors have to provide plug-and-play capabilities that enable to detect active sensors in a fast and accurate way.

In the last several years, intelligent sensor nodes have reduced in size (which is why they are also called motes), and, despite the current progress on energy harvesting techniques [22], they still depend on batteries to operate. Therefore, in order to minimize sensor node consumption, it is necessary to optimize the firmware of the deployed sensors and make use of efficient communication technologies and lightweight protocols, which are also able to reduce response latencies and improve Quality of Service (QoS).

There are currently in the market hundreds of IoT manufacturers that develop prototypes, solutions, and proprietary SDKs [23]. Moreover, in this diverse IoT landscape, there is a lack of spread of interoperability standards for all layers, from the physical to the application layer. This heterogeneity makes it difficult to integrate intelligent sensors from different manufacturers into an application or an IoT system. In addition, there is a growing Do It Yourself (DIY) community of makers that devise new products and customized IoT developments that are facing the mentioned challenges everyday.

In response to this need, the Web of Things (WoT) [24,25] arises as a concept that creates an abstraction layer above IoT, modifying the already existing web protocols that have provided scalability and interactivity to applications and that allow web developers to make use of the data and services collected from IoT devices. While IoT solves networking problems, WoT is focused exclusively on application layer protocols and tools [26]. The following are the main issues to be solved:

- There is a lack of spread of solutions that provide plug-and-play mechanisms at the sensor node layer.
- There is a need for standards to enable the interoperability between sensors of different manufacturers or that make use of diverse communication technologies.
- There is a need for human-centered tools to configure motes dynamically in order to reduce the development time, even allowing the DIY community to implement IoT applications with little or no knowledge about electronics or programming.
- There is a need for lightweight protocols that enable mechanisms of detection and self-registration for the communications performed between a mote and a gateway, between a gateway and a cloud, and between a mote and a cloud.

In order to tackle the previously mentioned issues, this article proposes a solution that includes the following main contributions:

- An architecture based on the IEEE 21451 standard that proposes different modifications related to the concept of Virtual Transducer Electronic Data Sheets (VTEDS) is presented. The proposed system allows for providing plug-and-play mechanisms at the sensor layer of an IoT ecosystem.
- A web application with an intuitive graphic interface that allows for monitoring, controlling, and managing all the sensors and the communications architecture is also presented.
- The proposed system is evaluated empirically in diverse real scenarios. The performed experiments make use of different sensor nodes in order to show that the designed architecture is very fast when deploying sensors and exchanging data.

The rest of this article is organized as follows. Section 2 reviews the most relevant interoperability initiatives, emphasizing the importance of IEEE 21451. Section 3 describes the main elements of the proposed VTED-based architecture. Section 4 details the implementation of the architecture. Section 5 describes the experimental setup and analyzes the results of the performed experiments. Finally, Section 6 is devoted to conclusions.

## 2. Related Work

### 2.1. About ISO/IEC/IEEE 21451

The development of IoT systems to create ubiquitous computing environments requires heterogeneity, scalability, interoperability, flexibility, reliability, and availability. To face such challenges, especially those on sensor heterogeneity and interconnectivity, it is necessary that sensors are designed according to standards widely accepted by the industry. In this context, one of the most relevant standards is ISO/IEC/IEEE 21451, which was initially named IEEE 1451 when it was first created by the IEEE Instrumentation and Measurement Society’s TC-9 Technical Committee on Sensor Technology. The aim of IEEE 21451 is to standardize the connectivity of smart sensors for different instruments and systems through a local network or through the Internet, easing the interoperability of devices and data [27].

**Figure 1:**
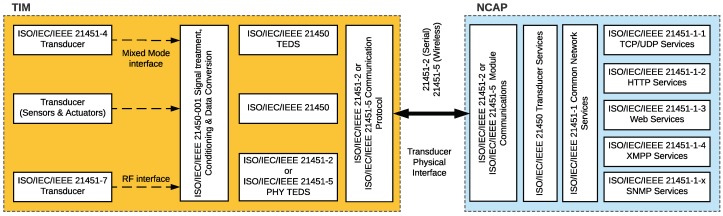
The IEEE 21451 family of standards and their relationships.

**Figure 2:**
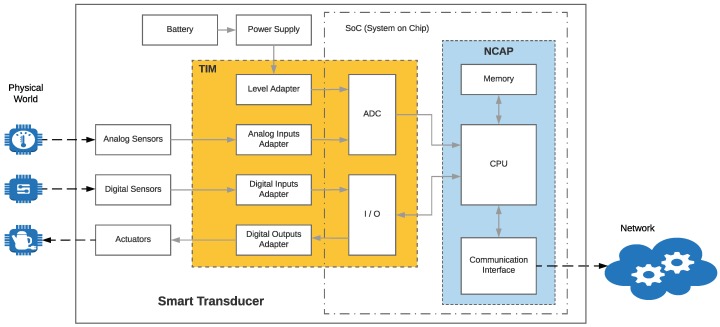
Smart transducer basic structure.

Actually, IEEE 21451 is a family of standards: IEEE 21451.0 defines common functionalities, commands, and TEDS; IEEE 21451.1 describes common network services; IEEE 21451.2 details serial interfaces (UART, USB, I2C, and SPI); IEEE 21451.3 defines web services; IEEE 21451.4 defines MMI (mixed-mode interface); IEEE 21451.5 standardizes the data format and the methods for providing wireless communications through technologies like IEEE 802.11x (WiFi), IEEE 802.15.1 (Bluetooth), IEEE 802.15.4 (part of ZigBee), or 6LowPAN; IEEE 21451.7 defines the interface for Radio Frequency Identification (RFID); and IEEE 21451.1.4 describes the interface for eXtensible Messaging and Presence Protocol (XMPP). Figure 1 shows a summary of the IEEE 21451 family of standards and their relationships. As can be observed in the figure, the standards are defined in relation to the two main components that comprise a smart transducer (the Transducer Interface Module (TIM) and the Network Capable Application Processor (NCAP)), whose basic internal structure is depicted in Figure 2.

The TIM is where sensors and actuators are located and acts as an interface with the actual instrumentation and with the automation processes. This module also processes the signal obtained from the sensors before sending it to the NCAP.

A TIM can contain both smart transducers (that embed TEDS) or conventional transducers, which currently represent the vast majority of the market. Despite the heterogeneity of manufacturers and standards, transducers can be classified into six groups depending on their interface: analog, digital, UART-based, 1-Wire-based, SPI-based, and I2C-based [28].

Regarding the NCAP, it is the network device responsible for communicating the smart transducer with the higher layers of the architecture, where cloud computing and user applications are located. In practice, it can be said that the NCAP acts as a sort of data communications gateway.

An intelligent NCAP can be characterized in order to create plug-and-play mechanisms and to detect and calibrate the connected transducers automatically, enabling interoperability and adaptability. Specifically, an NCAP should have the following features [28]:

- There should be standard physical and electrical connections between an NCAP and its transducers.
- The NCAP should be able to diagnose the state of a transducer.
- It should be possible for the NCAP to detect and identify a new transducer.

For ease transducer identification, IEEE 21451 proposes the use of TEDS, which are responsible for describing the transducers through a set of metadata that resemble the datasheets provided by the manufacturers of electronic components. Such metadata can include the device ID, the manufacturer’s name, the serial number, or the calibration data. IEEE 21451 provides a list of already defined templates that cover the majority of cases (some examples are given in Table 1), although it is possible for a user to define proprietary TEDS [29].

Initially, TEDS were embedded in the transducer memory and enabled basic plug-and-play functionalities, which required adding self-identification and self-description mechanisms. However, such a solution has certain limitations, since, in general, each transducer follows a specific standard, requiring complex hardware in the TIM and NCAP, which raises the cost to implement TEDS. In addition, the increase in the number of transducers also raises complexity and, as a consequence, the system’s cost. Moreover, adding, removing, or modifying a transducer requires non-automated processes and the need for an expert, which also increases the system’s complexity and cost.

A solution to the mentioned problems is provided by VTEDS, which allow for storing the information of the sensors in other devices of the network, either in the NCAP or in a remote dedicated server. This paper focuses on the use and advantages of VTEDS, but note that such a solution also has its limitations, since a TED library is required and some manufacturers do not publish their transducer TEDS.

#### 2.1.1. Previous IEEE 21451 Implementations

Some implementations of the IEEE 21451 standard have been developed during the last decade. For example, in [31], a system with plug-and-play sensors and auto-calibration that is compatible with the IEEE 1451.4 standard is implemented. Another interesting example can be found in [32], where the development and implementation of a plug-and-play system based on IEEE 1451.4 and aimed at collecting data from a temperature sensor is described. In such a system, the temperature sensor embedded TEDS into a DS2433 Electrically Erasable Programmable Read-Only Memory (EEPROM), while the NCAP was implemented in two separate modules: an 8-bit microcontroller and a LabView application. In [33], the implementation of a ZigBee network for lighting control based on IEEE 21451, where the TIMs are the lighting end-nodes and the required coordinators of the Zigbee network are connected via USB to a PC (both comprising the NCAP), is presented. The proposed architecture uses REpresentational State Transfer (REST) web services and a MATLAB program that runs on a PC to decode the received metadata. Another application based on ZigBee is presented in [34], where the combination of a ZigBee coordinator and a PC that runs LabView to decode the TEDS metadata is used as an NCAP.

The authors of [35] implemented an IoT solution using VTEDS and IEEE 21451.4 commands for ModBus devices. Another example is described in [36], where the use of the IEEE 21451-001 standard in the processing of signals from smart transducers is demonstrated. The proposal also defines its own segmentation algorithm for signal processing and a table of algorithms called the Transducer Algorithm Data Sheet (TADS). The segmentation algorithm is simulated in MATLAB, so it is recommended to be executed on microprocessors, since it requires a relevant amount of resources that cannot be provided by many motes.

In addition, in [37], an IEEE 21451-based system for monitoring air quality using GSM wireless communication modules is proposed. In contrast, a more generic system is presented in [29], where the authors use Python to create a platform independent of TEDS based on the IEEE 1451.0. In the proposed system, an Arduino [38] was used as TIM and a Raspberry Pi [39] acted as an NCAP.

An interesting initiative to incorporate semantics into wearables while implementing the IEEE 21451 standard is presented in [40]. Although the described solution was only simulated, it clearly demonstrates how smart sensor representation can be performed at the semantic level. Similarly, in [41], a solution that defines an ontology to describe the sensors and that establishes a link between the IEEE 1451 specifications and the operations of Open Geospatial Consortium (OGC) Sensor Observation Services (SOSs) is presented.

Finally, in [42], the authors present a Home Automation System (HAS) implementation using intelligent transducers with plug-and-play capabilities based on IEEE 21451 TEDS. The system makes use of a REST Application Programming Interface (API) to access the gateway to obtain the data from the registered sensors. Since the use of traditional GET/POST requests introduces latencies into the system, the researchers propose the use of a Publish/Subscribe (Pub-sub) messaging system.

### 2.2. OGC Standards

OGC brings together several public and private organizations, responsible for defining open and interoperability standards, mainly for Geographic Information Systems (GIS) and the web. The next subsections analyze some of the OGC standards related to telemetry applications that allow sensor description and interoperability.

#### 2.2.1. OGC-Sensor Web Enablement Framework

Sensor Web Enablement (SWE) [43] is a relevant alternative to IEEE 21451 that has been standardized and used by the scientific community to provide interoperability. Specifically, it is developed and maintained by OGC with the objective of enabling the discovery, exchange, and processing of sensor data [44,45].

SWE consists of an architecture formed by a set of standards that defines the data format similarly to a web service interface [46]. Among the OGC standards, the following are some of the most relevant:

- Observations & Measurements Schema (O&M): it defines a scheme for encoding sensor data.
- Sensor Model Language (SensorML) and Transducer Markup Language (Transducer ML or TML): they allow for describing sensors.
- Sensor Observations Service (SOS) and Sensor Planning Service (SPS): they provide the link between the user and the measurements of the sensors.
- Sensor Alert Service (SAS): it defines the publish and subscribe scheme to send sensor alerts.
- Web Notification Services (WNS): it allows for delivering messages or alerts from SAS and SPS to users.

OGC also maintains the OGC Interoperability Program (IP) and OGC Specification Program and its OGC Network, an on-line Internet access infrastructure that implements the OGC standards.

**Figure 3:**
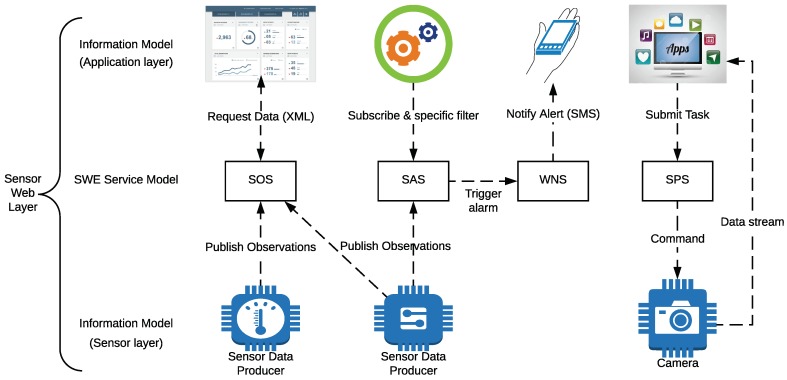
Example of Open Geospatial Consortium–Sensor Web Enablement (OGC-SWE) infrastructure.

As an example, Figure 3 shows an OGC-SWE architecture that allows for identifying the different layer levels of applications and sensors. Such a system enables sensors to be accessible and controllable through the Internet.

SWE has also been effectively implemented over IEEE 1451 NCAPs, which resulted in a high level of interoperability between sensors on sensor nodes and among sensor nodes inside a network [45]. Another example can be found in [47], where a ubiquitous sensor network platform for integrating smart cars is implemented, which includes several OGC-SWE services. However, the authors warn of problems in response times when large repositories are used and rapid reactions are required.

Meteorological approaches have also been proposed for SWE. An example is presented in [48], where the implementation of a network of hydrometeorological sensors based on SWE is described. The proposed model sends the information of the sensors in an O&M scheme to describe observations and their measurements. In addition, SensorML is applied to describe sensors, and SWE Common is used to send common data of the SWE and TML services.

Another example of using the SWE model to implement IoT applications related to topological or geospatial signals can be found in [49]. Such a work shows one of the shortcomings of OGC-SWE: it makes use of a REST architecture that includes a specific latency for telemetry applications. In contrast, the proposed solution based on VTEDS makes use of MQTT with a publish/subscribe architecture, providing the system with flexibility and interoperability.

#### 2.2.2. OGC-SensorML

Sensor Model Language (SensorML) [50] is a general model and XML coding scheme for sensors and observation processes. SensorML, besides being part of the OGC SWE framework, can also be used independently. The main function of SensorML is the definition of observation processes based on the collected measurements. For such a purpose, it provides descriptions of the sensors, actuators, and filters such as their geoposition, the observed values (measured data), or their accuracy, among others. It also defines an executable process chain for deriving observations.

In SensorML, all components are modeled as processes. Sensors and actuators are regarded as process components and the platforms are modeled as systems. All such components can participate in process chains, which are processes with inputs, outputs, and parameters.

There are two large groups of processes: physical processes, which are formed by detectors, actuators, and sensor systems, and non-physical or “pure” processes, which can be handled as mathematical operations.

**Figure 4:**
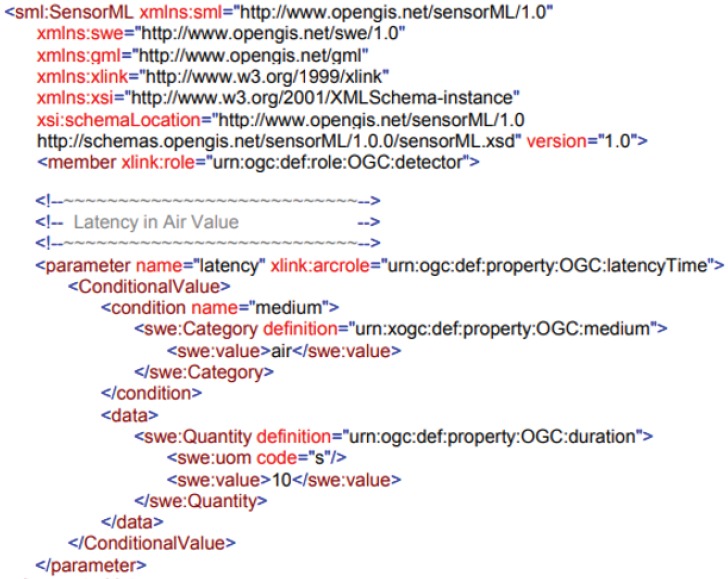
Example of XML for defining a sensor latency value.

The models in SensorML are encoded as XML schemas. Therefore, XML instance documents are created for all components and observation processes. Figure 4 shows an example of XML adapted from [50], where a latency of 10 s is defined to a specific sensor.

#### 2.2.3. OGC-PUCK Protocol

The OGC Programmable Under Water Connector (OGC-PUCK) [51] standard defines a protocol to be used in instruments with RS-232 and Ethernet communication ports. Like SensorML, the PUCK protocol belongs to the OGC-SWE suite, and it is mainly used to store and retrieve the description of an instrument. Nonetheless, it can also be used independently from the other SWE standards. An interesting feature is that PUCK can include the IEEE 21451 TEDS described in Section 2.1 as descriptors. Thus, PUCK is able to make use of descriptive documents in OGC-SWE SensorML or IEEE 21451 TEDS format as a “driver” code for an instrument.

A client that can interpret the PUCK protocol can then automatically retrieve the instrument metadata. Typically, both the SensorML and the instrument controller code will be stored in the PUCK memory of the instrument before implementation. When the instrument is powered on, the host recovers the metadata to identify the instrument. Such an identification process is called plug-and-work and is described in the next subsection.

#### 2.2.4. OGC-Plug-and-Work Mechanism

**Figure 5:**
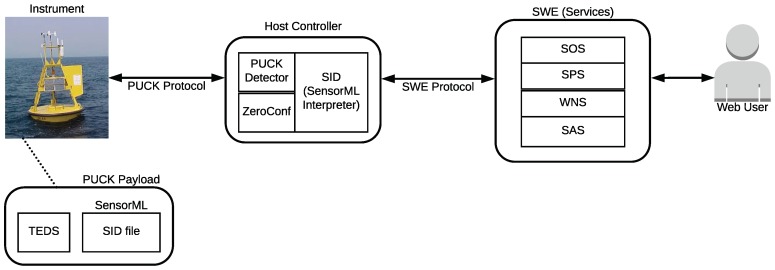
Plug-and-work architecture using OGC–Programmable Under Water Connector (PUCK) and Sensor Interface Descriptor (SID).

Figure 5 summarizes the flow and the set of OGC elements and standards involved in the OGC plug-and-work mechanism [52], which is the process to detect and identify an instrument to integrate it automatically into an observing system when the instrument is powered on. Then, a web client can retrieve the observation data via the Internet.

This mechanism begins at the instrument level with the modeling of the instrument through the Sensor Interface Descriptor (SID), which is based on the OGC SensorML standard, through which the protocols and data format of each instrument are described. Then, such descriptions (SID files) are stored in the instrument’s memory itself through the PUCK payload, which includes both TEDS and SID files (XML files), as can be observed in Figure 5. Then, at the host level (Observation System), modules are implemented so that a PUCK-enable instrument can be detected. The PUCK detector module is used at the RS-232 level, which can detect the baud-rate of the RS-232 serial communication, while the ZeroConfig module allows for detecting IP-based instruments. Once an instrument is detected through the SID interpreter, all the metadata of the sensors are extracted and parsed from their XML files. Therefore, the SID interpreter acts as a gateway. Finally, the retrieved observation data is available at the web level, through the set of web services (SAS, WNS, and SPS) available through the SWE standard. Thus, the combination of PUCK with SID enables plug-and-work capability. More details on the OGC standards and the OGC plug-and-work mechanism can be found in [53].

### 2.3. Other Initiatives for Providing Plug-and-Play and Interoperability

There are other initiatives that do not make use of IEEE 21451 or SWE but that have contributed to the development of IoT by adding mechanisms that enable interoperability. For instance, a plug-and-play mechanism is presented in [54], where hardware modules (not the transducers themselves) are identified. Each module has a hardware descriptor, which implies that, if new sensors are added to the node, then such descriptors must be modified off-line so that the main board software can describe them.

Another solution that provides plug-and-play functionality through hardware that acts as a peripheral hub is described in [55]. In this paper, the peripherals are identified by using a network of passive electrical components (resistors). Once the modules physically connected to the hub have been detected, the application, developed on Contiki [56], asks the gateway for the necessary drivers to identify and make use of the connected peripherals. The drivers are generated with a proprietary language that is able to implement complex processes. The detection of hardware peripherals is a significant limitation of this solution, as well as the complexity of its implementation.

The authors of [57] introduce a lightweight model for semantic annotation of data using heterogeneous devices in health applications. To achieve a semantic interoperability, they use a Resource Description Framework (RDF) [58] for the representation of the data, and SPARQL to read them.

In [59], an architecture to provide interoperability to IoT systems taking platforms, things, users, and developers into account is introduced. It is also worth mentioning the project detailed in [60], which, although in a pilot stage, is an important initiative in achieving the desired abstraction of hardware platforms, application domains, and services. Finally, another excellent work [61] shows the evolution and challenges that interoperability has had in relation to IoT, WoT, and the Semantic Web of Things (SWoT) [62]. In the paper, the authors detail a case study of the SWoT in the European project FIESTA-IoT [63], where a semantic engine to annotate WoT data and an SWoT generator in the context of OpenIoT is implemented [64].

After reviewing the state of the art and in light of observing an evolution from TEDS toward VTEDS, it can be concluded that most of the papers present closed systems that depend on certain manufacturers who do not usually provide their TEDS to third parties. Such a dependence derives into expensive solutions and high-complexity implementations that are often aimed at solving very specific problems of an application domain. Some of these proposals provide solutions with an NCAP generally connected to a PC or server via USB, limiting the sensor’s application domains. In those cases, the NCAP’s computational load related to the management of the TEDS is very high (for example, it may require the use of a desktop computer that runs LabVIEW or Matlab), which restricts the use of the less powerful processors and microcontrollers, which are the ones usually embedded into current IoT smart transducers mainly due to the use of batteries.

In addition, there is a need for a flexible abstraction layer that would allow for the description of any hardware, regardless of the manufacturer or technology, and that supports lightweight third-party communication protocols, semantic descriptions, and a simple, agile and low-cost deployment of the current heterogeneous transducers.

To tackle the mentioned issues, this paper presents the design and development of a plug-and-play architecture based on VTEDS with self-description at the sensor layer and where VTEDS are available at the cloud layer in order to ease transducer management from the web and to let other applications and services use the information provided by the IoT system.

### 2.4. Analysis of the Related Work

After reviewing the related work, it is possible to carry out a comparative analysis of the solution proposed in this paper and the previously mentioned standards, emphasizing the advantages of the new proposal.

First, it is important to note that both the IEEE 21451 and the OGC standards provide options that guarantee sensor interoperability, including the implementation of plug-and-play mechanisms like the ones previously described. Such standards make use of similar concepts and resources, making it possible to combine them. For instance, TEDS can be used both by IEEE 21451 and OGC-based solutions to describe the metadata of the sensors. Similarly, the solution presented in this paper, which is based on the IEEE 21451 standard, uses the same meta-descriptor (TEDS) as the OGC-PUCK standard. Another similarity lies in the use of a protocol to encapsulate the descriptors: in this paper, the Lightweight Protocol for Sensor (LP4S), which is similar to OGC-SensorML, is used.

Nonetheless, both OGC-PUCK and IEEE 21451 implementations often store the descriptor into the sensor or instrument, which can pose a problem when updating the information. This is resolved through VTEDS, which add the possibility of using repositories for external SID files. The SID files used as meta-descriptors must be created before the implementation of the solution. The creation of SID files without tool support can be a tedious and error-prone process. An example of a visual SID Creator application is found in [65]. In contrast, in the proposed VTEDS system, all system parameters are created through a web interface accessed through the Internet, enabling its immediate use. Whenever VTEDS change, the system generates publications in the corresponding topic, in such a way that the sensor node consumes the performed updates in real time, through the mechanisms that will be explained later in Section 3.3. An additional advantage of the presented approach is that it can reuse the descriptors available in the cloud among different domain applications when necessary, thus updating and adding new VTEDS in a transparent way for the sensor level.

Regarding the OGC-PUCK solution, it has two significant limitations when it comes to the implementation of modern telemetry projects based on power-constraint wireless sensor nodes. The first limitation is the amount of memory and processing resources needed to deploy the plug-and-play mechanisms when the PUCK payload is embedded into the sensor node. Sometimes, the PUCK payload has to be saved on an SD card as in [66]. Furthermore, OGC-PUCK is not optimized for being used in power-constraint devices with low memory and processing. For instance, OGC-PUCK requires to include drivers to manage RS-232 or Ethernet ports. This issue is tackled by the proposed system with the use of the LP4S protocol, which can be embedded even in resource-constraint hardware devices like Eddystone beacons (more information on the LP4S protocol can be found in [67]).

The second disadvantage is that there are significant delays in hardware detection when obtaining measurements. For example, in the case of PUCK-RS232, the PUCK software performs a cyclic process to synchronize the speeds of the serial port of the instrument with the host, which derives into additional latencies. A similar process occurs in the Zeroconfig module for IP-PUCK instruments. Another delay arises when the mechanism used to obtain the observation data retrieves the sensor data from an SID file. Both processes are well explained in [53]. In comparison, the proposed solution based on VTEDS solves such a limitation quickly and efficiently, as is shown in Section 5.

## 3. Plug-and-Play Architecture Based on Virtual TEDS

This section presents the general architecture of the proposed system and describes a novel plug-and-play mechanism. As will be detailed in the next subsections, the IEEE 21451 standard was chosen to describe the sensor hardware with an adaptation on the use of VTEDS.

### 3.1. Global Overview

**Figure 6:**
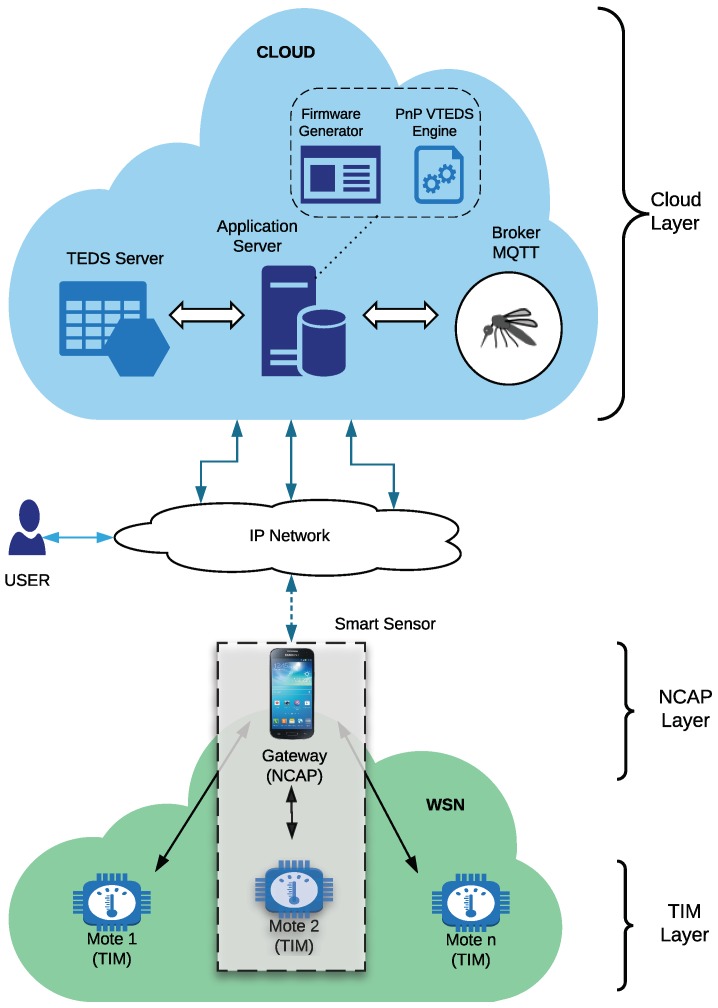
Plug-and-play architecture based on Virtual Transducer Electronic Data Sheet (VTEDS).

Figure 6 shows the proposed architecture, where the main components defined by IEEE 21451 are included. The different components are grouped into three layers: TIM, NCAP, and Cloud layers.

The sensor nodes or motes are the TIMs of the system, which form a WSN. TIMs are in charge of monitoring the environment. For example, a temperature or a humidity sensor can send the data read to the cloud through the NCAP, which acts as an IoT gateway that processes and then sends the collected data to the cloud. The NCAP, in most cases, forwards the collected data into an IP network, so it is often necessary to perform adaptations on the different protocols and communication technologies.

The server that hosts the VTEDS is also in the cloud, where there are also IoT applications responsible for generating the firmware for the sensor nodes and the services needed to discover automatically the active TIMs. The database of VTEDS hosted in the cloud is available via web services for both the monitoring applications of the system and for any other IoT application that requires it.

### 3.2. Messaging System and Communication Protocols

In the last years, different messaging protocols have been proposed to interconnect heterogeneous IoT nodes. Traditionally, the heterogeneity problem was solved by implementing file exchange services, deploying shared databases, or using Remote Procedure Call (RPC) mechanisms, but the latest developments were aimed at creating messaging systems.

Messaging systems centralize communications to minimize the coupling between the components of distributed systems like the ones usually deployed in IoT developments. This decoupling and the use of asynchronous communications allow for carrying out more robust communications: the components to be communicated do not have to be working at the same time, so they delegate the delivery of the data to a messaging system, which enables them to focus on which information to send instead of how to send it.

Most messaging systems make use of two models for connecting a transmitter and a receiver: the peer-to-peer queue model or the publish/subscribe model. The peer-to-peer queue model enables the transmitter to send a message to a queue where it will be read by a particular receiver. In the case of the publish/subscribe model, messages are sent to one or more recipients who have previously expressed interest on them. In practice, when the transmitter sends (publishes) a message, it is actually sent a copy to a receiver (subscriber). Examples of popular messaging systems (pubsub) include Java Messaging Service (JMS) (e.g., OpenJMS, WebSphereMQ), Advanced Message Queuing Protocol (AMQP) (e.g., Apache Qpid, SwiftMQ, RabbitMQ), XMPP (e.g., Ejabberd, Apache Vysper), and Message Queuing Telemetry Transport (MQTT) (e.g., ActiveMQ, Mosquitto).

MQTT [68] is specially interesting for IoT, since it has been designed as an open messaging protocol that enables exchanging messages with resource-constraint devices (i.e., sensors, actuators, and mobile phones) that operate in high latency networks. For these reasons, MQTT is very popular among IoT developers, who have used it in smartwatch applications [69], robotics [70], home automation [71], and healthcare [72].

Due to the previously mentioned advantages, in the system proposed in this article, at the cloud layer, all the messages that come from the WSN are managed by an MQTT server (broker) that receives asynchronously the data from the remote sensor nodes.

The messaging system, both on the side of the TIMs and the NCAPs, makes use of the LP4S family of sub-protocols (LP4S-6, LP4S-X, and LP4-J), which are aimed at providing lightweight communications to battery-operated and resource-constraint IoT devices. The protocols developed as part of a previous work are described in detail in [67], and their main features include the following:

- They can be embedded into Bluetooth Low Energy (BLE) beacons.
- They can send data collected from beacon sensors and receive commands for actuators.
- The LP4S family of protocols requires little memory and CPU usage, which allows it to be implemented in low-resource devices. The protocols also reduce communication time, thus decreasing power consumption. For instance, the LP4S protocols were tested with Generic Attribute Profile (GATT)-based beacons, yielding a power consumption of only 35 μA in standby mode (before connecting to other devices) and an average of 575 μA when one or more users connected occasionally to the mote. In the case of the LP4S-6 protocol, it was tested on an Eddystone beacon with 100 ms to 5 s beaconing intervals, obtaining power consumptions between 125 and 865 μA, which suggests that such a protocol may be used in scenarios where low-energy consumption is essential.

### 3.3. Plug-and-Play Mechanism

This section describes the processes that have to be run on the TIM, NCAP, and cloud layers to create dynamic descriptors of the sensors/actuators based on the information they send, which allows for the discovery of the installed hardware (i.e., the number of connected transducers and their nature).

#### 3.3.1. Auto-Configuration and Self-Registration at the NCAP and TIM Layers

The first plug-and-play mechanism of the proposed system starts at the TIM layer, where the installed hardware is first recognized (i.e., when attaching it to the TIM inputs/outputs). This process should be dynamic and flexible; therefore, the firmware of the TIM has to be designed to preserve such a dynamism and flexibility.

**Figure 7:**
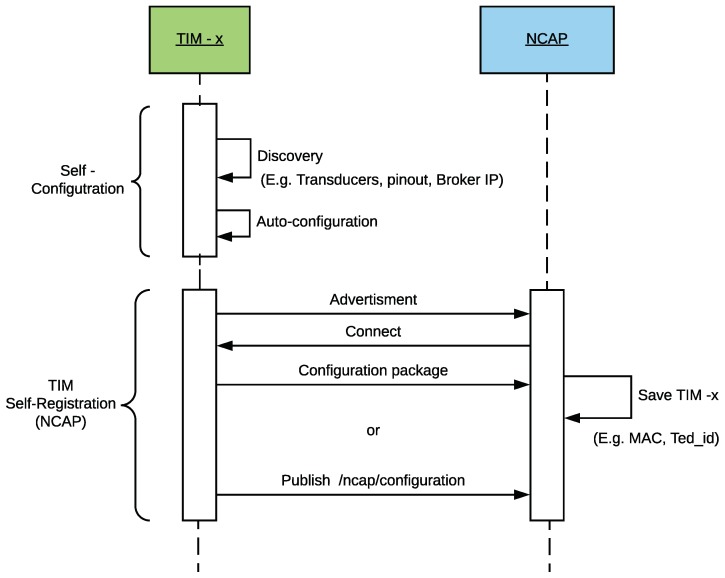
Self-configuration and self-registration of the Transducer Interface Module (TIM) at the Network Capable Application Processor (NCAP) layer.

Figure 7 shows a sequence diagram where the TIM is first self-configured and then self-registered at the NCAP layer. The term self-registration means that the TIM is capable of communicating its presence and characteristics to the higher layers by itself.

Hardware discovery in the TIM can be performed thanks to a structure of descriptors (labels) that indicate its characteristics: the location of each pin, whether a pin is analog/digital or an input/output, the embedded communication modules, or the network configuration (connections to other equipment, IPs, ports, or delays). The values associated with the descriptors can be modified at any time without altering the firmware core. In this way, a user without programming experience can configure a TIM by only assigning a value to each of the descriptors. Therefore, when the firmware of the TIM reads the descriptors, it discovers the operating parameters and performs the self-configuration procedure. This process occurs every time the TIM is restarted.

Once a TIM is self-configured and ready for operation, it is necessary to inform the upper layers (NCAP and cloud) of its configuration, so that remote users can access the TIM from such layers, either to monitor the values of the sensors or to send a command to the actuators. Thus, the TIM must be first registered in the NCAP layer. The process is dynamic and transparent for the end user, since the TIM is capable of self-registering in the system, making use of the LP4S protocol. In this way, the TIM sends an LP4S configuration frame automatically which can be used by the NCAP to extract all the parameters of the TIM and to store them in their database (what will speed up the subsequent accesses to the TIM). This process is called self-registration.

#### 3.3.2. Self-Registration and Auto-Calibration at the Cloud Layer

**Figure 8:**
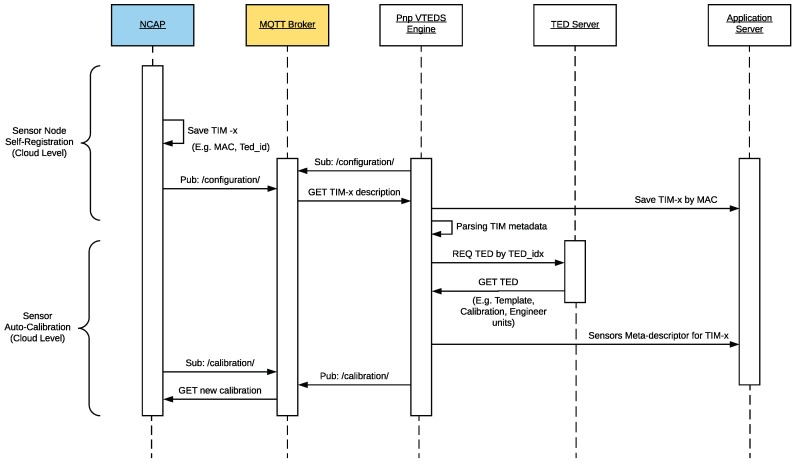
Self-registration and auto-calibration of the sensor node in the cloud.

The sequence diagram on Figure 8 illustrates the self-registration and auto-calibration processes of a sensor node in the cloud. The process begins when the NCAP, using the LP4S-J protocol (previously mentioned in Section 3.2), sends a JSON to the cloud indicating the TIM configuration parameters. In fact, the NCAP publishes the TIM information on the cloud MQTT broker, where the services (Pnp VTEDS Engine) and applications of the system are subscribed to the configuration topic. Thus, TIM metadata are extracted from the JSON and stored in the server database. This process is repeated for each of the TIMs associated with every NCAP.

Regarding auto-calibration, it allows for calibrating every sensor registered in the system in a dynamic and transparent way for the user. Therefore, the need for sending a technician to the location of the sensor node is avoided. This is especially useful when the nodes are installed in remote places.

The parameters for sensor calibration are entered into the system through a web browser. The web application saves every calibration value in the database, so it is possible to restore in the future old calibration values.

The new calibration values are also managed by a service of the system; therefore, once a user requests a change of the calibration settings, the system publishes such a request in the MQTT queue of the calibration topic associated with the MAC of the TIM to be calibrated. Moreover, the NCAP is subscribed to the calibration topics of the discovered TIMs.

### 3.4. Metadata of a Sensor Node

**Figure 9:**
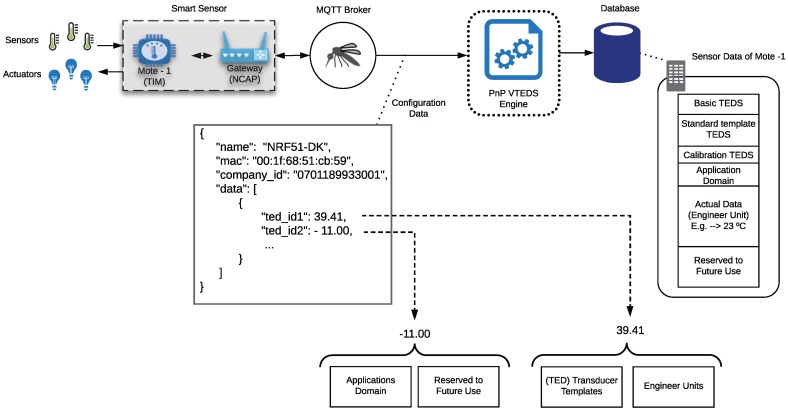
Metadata structure and collection from a smart sensor.

The metadata of a sensor node contains a set of descriptors that include some related to IEEE 21451 and some additional VTEDS that allow for creating new descriptive and semantic implementations. The metadata stored in the cloud database is on the right of Figure 9, and it complies with the structure proposed by IEEE 21451.

Figure 9 shows a JSON that contains the structure of the sensor node metadata stored in the system database. On the example illustrated in Figure 9 three basic TEDS are shown: “name” (the name of the sensor node), “mac” (unique MAC address of the device), and “company_id” (proprietary company id of the manufacturer). There are two additional descriptors (“ted_id1” and “ted_id2”) that enable including additional information on the sensor node (e.g., semantic descriptions).

Figure 9 also shows two VTEDS: the first one (39.41) describes a standard template together with the measurement unit, while the second one (−11.00) defines the application domain. Specifically, TED_id1 (39.41) describes a sensor through a standard template (type 39, a potentiometric voltage divider) and indicates that the unit of measurement can be found in Index 41 on the unit of measurement table (for instance, such a unit can be millivolts).

In TED_id2 (−11.00), the field −11 may represent, for instance, the *Precision Agriculture* application domain. The negative sign was included only to show that negative values are also allowed on the descriptors. The fields within the JSON change dynamically, according to the sensors and actuators connected to the TIM, and there can be as many TED_id fields as necessary to describe a transducer.

It is important to note that the TED_id field is represented by using an 8.8 fixed-point format. That is, an 8-bit signed integer followed by an 8-bit unsigned integer. Therefore, in the previous example, TED_id1 can define up to 128 templates and 256 values. Such a fixed-point format is actually related to the use of the LP4S protocol, which is optimized for hardware architectures constraint in terms of memory, CPU, and other resources (e.g., BLE beacons) [67].

### 3.5. Sensor and Actuator Data Flow

**Figure 10:**
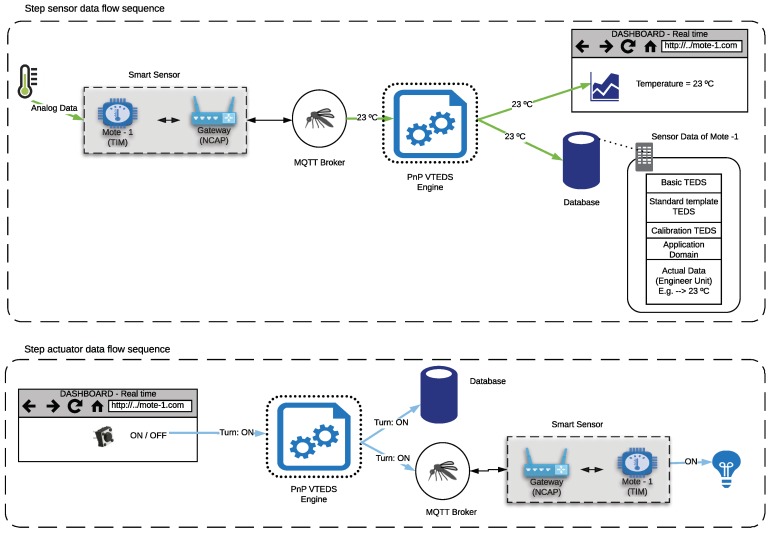
Data flows between sensors/actuators and Internet of Things (IoT) applications.

Figure 10 depicts the operation data flow in the system from the point of view of a sensor (in Figure 10, at the top) and an actuator ( (in Figure 10, at the bottom)). In the case of the sensor, it is assumed that it was previously discovered by the system, so the corresponding metadata were created in the database. Figure 10 shows the use of two specific transducers: an analog sensor and a digital actuator. The analog sensor is depicted with the icon of a thermometer, and its data flow describes the publication of the real-time temperature value on the MQTT broker. Such values are consumed by the plug-and-play mechanism in order to update the cloud database. In Figure 10, the obtained temperature (23 °C) can be read by a remote client through a web dashboard.

Regarding the data flow of the actuator, it starts in the dashboard, where there is a button to toggle the actuator’s state. When the user presses such a button of the dashboard, the system publishes on the MQTT broker the “Turn On” command. Such a command is consumed by the PnP VTEDS engine in order to update the database. The MQTT message is also consumed by the NCAP, which transfers it to the TIM that controls the actuator. Eventually, the TIM processes the NCAP message and activates the corresponding output, thus turning the desired actuator on.

### 3.6. Calibration Data Flow

Current sensor manufacturing technology provides high-resolution and accurate sensors, but their precision and repeatability (i.e., ideally, a sensor should always produce the same output for the same input) are affected by noise, hysteresis, linearity or change speed. For this reason, in telemetry it is very important to calibrate sensors in order to improve data accuracy.

Calibration consists in a set of operations that allows for establishing, under certain conditions, the relationship between a sensor output and the actual values sensed by an accurate measurement device. Such a relationship is usually modeled as a function, which can include, for instance, linear, exponential or logarithmic factors. Thus, the main way to establish this relationship consists in using correction tables or calibration equations (e.g., straight lines or curves). For example, in the case of straight lines, calibration can be performed with the correction of a single point (usually offsetting the origin) or with two calibration points, which changes the slope of the line. In the case of sensors that do not follow a linear response curve, the adjustment is often carried out through polynomial curves. Further details and a good introduction to the calibration process can be found in [73].

As a demonstration of the capabilities of the proposed system, its first version is able to make use of linear correction equations. However, it would be easy to add any other equation or technique depending on the used sensors.

**Figure 11:**
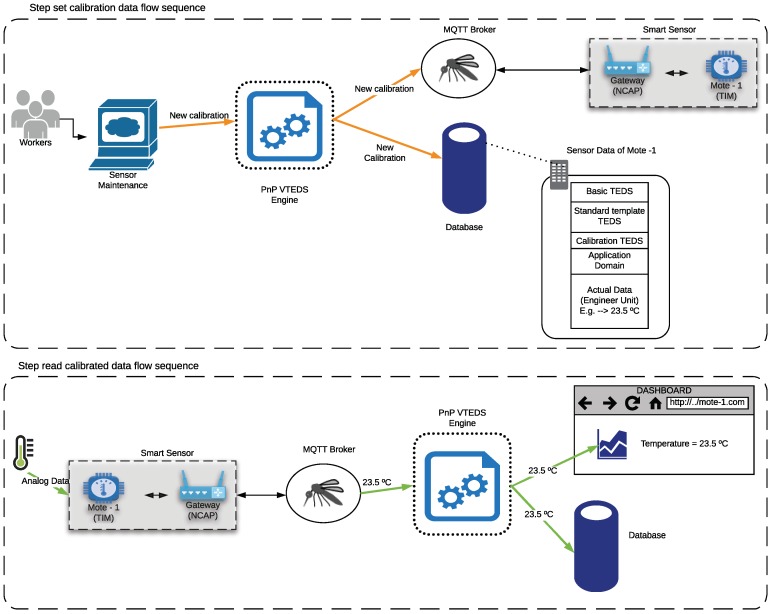
Data flows for new calibrations of sensors.

Figure 11 shows the flow diagram of the calibration procedure. The flow (step set calibration data flow sequence) begins when a user changes the calibration parameters of the temperature sensor of the previous example. Such parameters are modified in the metadata of the sensor in the database and publishes them in the MQTT calibration topic by the Pnp VTEDS engine. The flow ends in the smart sensor that is listening in the same MQTT topic, to self-calibrate. In the next reading of the sensor, the temperature value would be adjusted by the calibration parameters (step read calibrated data flow sequence). Therefore, the calibration of the sensor can be performed remotely and automatically, avoiding the need for carrying out in-situ calibrations by technicians and providing flexibility and dynamism to the IoT system that implements the proposed architecture.

## 4. Implementation of the Architecture

The previously described plug-and-play architecture has to be deployed on the three mentioned basic layers (TIM, NCAP, and cloud). In the next subsections, the implementation of such layers will be detailed in such a way that they can be reproduced by third parties using off-the-shelf hardware devices and commercial software tools.

### 4.1. The TIM Layer

**Figure 12:**
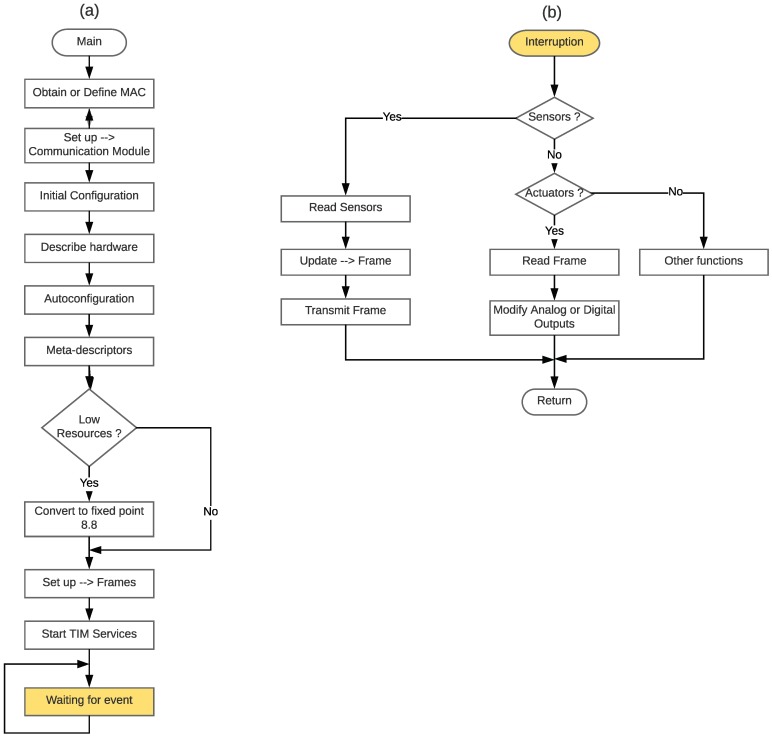
TIM flow diagrams: (**a**) Main program and (**b**) Interrupt Service Routine (ISR).

There is currently a large number of commercial devices, with different resources (communication interfaces, CPU, and memory) that can act as a TIM and on which a developer can implement a firmware according to Figure 12. For instance, there are several versions of Arduino that provide Ethernet connectivity or different devices that include BLE interfaces (e.g., RedBearLab nRF52832 [74]).

The data flow of the main TIM program is shown in Figure 12a and includes calls to methods that enable the discovery of the installed hardware. The use of intuitive and simple descriptors allows the TIM to self-configure and be ready to manage the sensors and actuators present in its digital and analog inputs and outputs. In addition, the firmware must include functionality to create meta-descriptors based on the potential hardware to be used. In the case of devices with reduced memory or CPU, it is recommended that the TED_id represents real numbers in the 8.8 fixed point format. After the initialization, the TIM sends a configuration frame to the upper layers of the system (i.e., to the NCAP and to the cloud) to perform the self-registration. Once this is achieved, the sensors can be remotely monitored from the web and TIMs are capable of receiving remote commands for their actuators.

It is recommended that the sensors and actuators be managed through interruption routines for better TIM performance. Figure 12b shows the Interrupt Service Routine (ISR) that must be implemented for real-time management.

### 4.2. The NCAP Layer

The other important element of the plug-and-play mechanism is the NCAP, which not only acts as the WSN gateway but also performs certain computing tasks. Depending on the used TIMs, their communication interfaces, processing power, and required memory, adequate hardware must be selected for the NCAP. Both commercial and proprietary solutions can be used. Some of the commercial hardware that has been tested as an NCAP are different SBCs (e.g., Raspberry Pi [39], BeagleBone [75], and Orange Pi PC [76]) and several Android-based smartphones [77].

**Figure 13:**
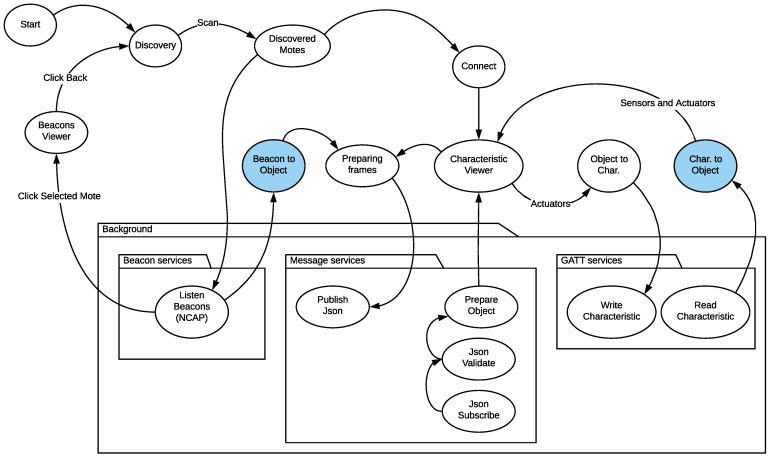
The NCAP basic state machine.

The degree of complexity of the implementation of an NCAP may differ depending on the selected hardware, the IoT application domain, the need for a physical display, database requirements, the local processing power, or the communication interfaces, among others. However, Figure 13 could be a good starting point for the development and programming of the NCAP. In the figure, a reduced state machine for an NCAP implemented on an Android smartphone that runs an application to manage BLE beacon-based TIMs is depicted.

### 4.3. The Cloud Layer

The third important element for the implementation of the proposed plug-and-play architecture based on VTEDS is the cloud, where TEDS will be stored and virtualized. The cloud, like the TIMs and NCAPs, can be commercial or proprietary. Examples of commercial solutions are Amazon AWS [78], Microsoft Azure [79], and Google Cloud [80]. In this article, it was decided that the the IotM@ch cloud platform [81] developed by the AutoMathTIC research group of the Technical University of Machala (Ecuador) in conjunction with the Group of Electronic Technology and Communications of the University of A Coruña (Spain) would be used. The platform provides features and functionality similar to other commercial IoT platforms, so it was mainly selected due to two factors: availability and cost. On the one hand, the IotM@ch platform was completely accessible, with total control over the hardware and software of the cloud infrastructure. It is also possible to manage the bandwidth of the local server, which allows for increasing performance and reducing application latency for the Internet remote clients. On the other hand, the Technical University of Machala had already installed server infrastructure, with the possibility of using the university’s resources without incurring in additional hardware costs and without needing to hire and train technicians dedicated to manage servers, access control, air conditioning, electric powers, or energy backup subsystems. In addition, note that, due to the number of services and applications deployed by the proposed system, which require the use of 5–8 virtual machines, the monthly cost for other IoT platforms like Google IoT usually ranges between $2000 and $3000, which is prohibitive for many universities in developing countries. For example, a monthly estimated cost of $2372.35 was obtained through the Google Cloud Platform Pricing Calculator [82] with the following parameters: Computer engine: 1 × Data analysis (n1-standard-4); Operating, System/Software: Free (Debian, CentOS, CoreOS, Ubuntu, or other user Provider OS), 1 × Big data (24 h per day), 1 × APP Server (n1-standard-2, SSD space 2 × 375 GB), 1 × Real-time monitoring (n1-standard-2), Cloud Storage (4096 GB), Cloud SQL Second Generation (one instance, 500 GB of storage, 500 GB for backups), and IoT Core (Data exchanged: 102,400 MB).

**Figure 14:**
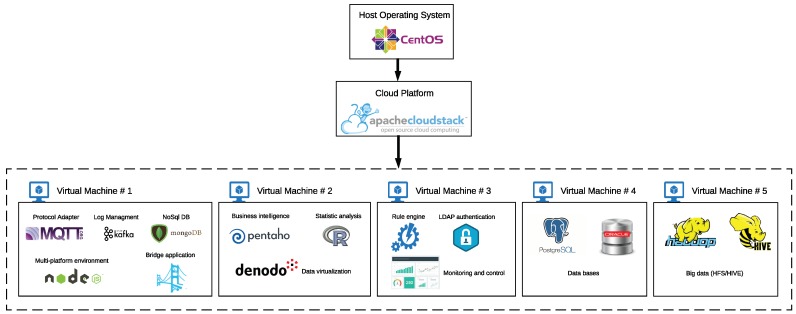
The IotM@ch cloud platform.

Figure 14 shows the most relevant elements of the cloud platform. Among them, the monitoring web application in Virtual Machine #3, which allows the management of both the discovered sensor nodes and the VTED server, is worth noting. In the next sections, the main applications that run on the cloud platform are detailed.

#### 4.3.1. TED Management

**Figure 15:**
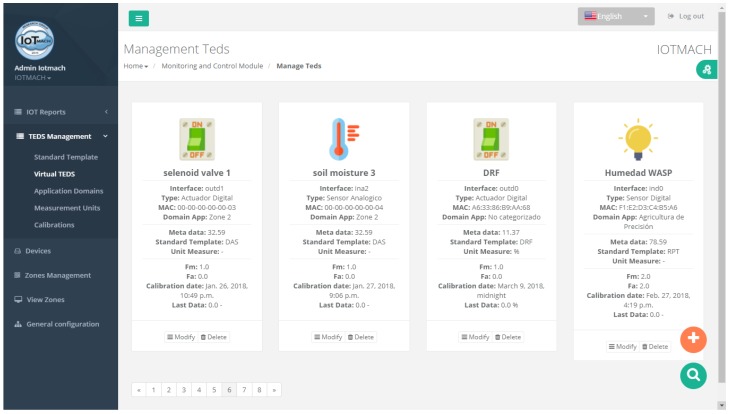
TED management application.

**Figure 16:**
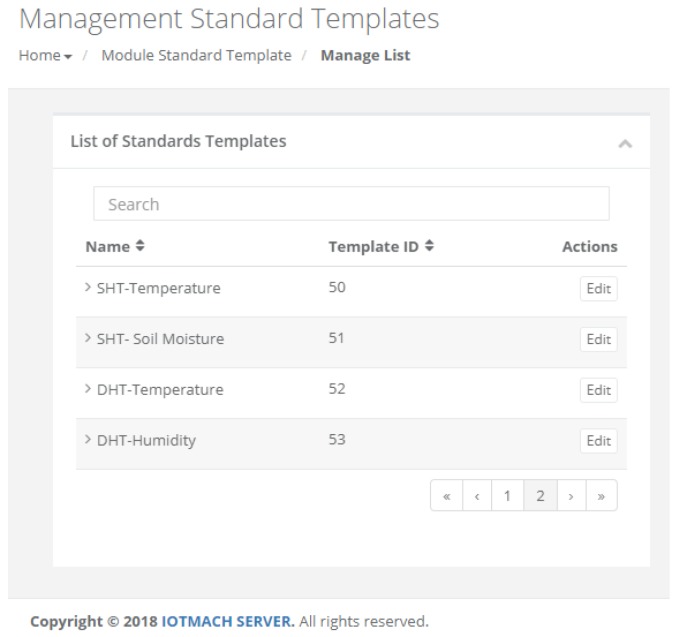
Standard template menu.

**Figure 17:**
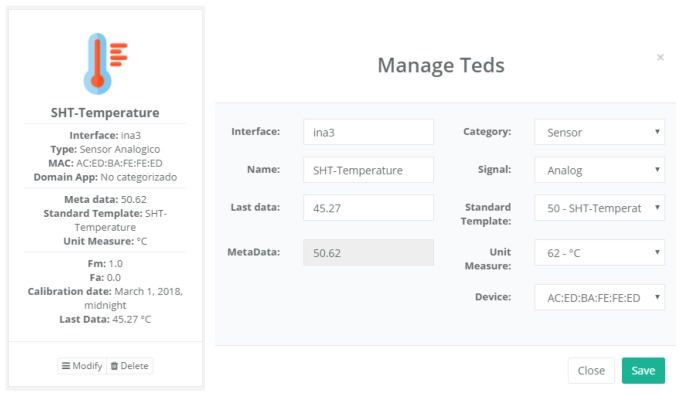
VTED management menu.

**Figure 18:**
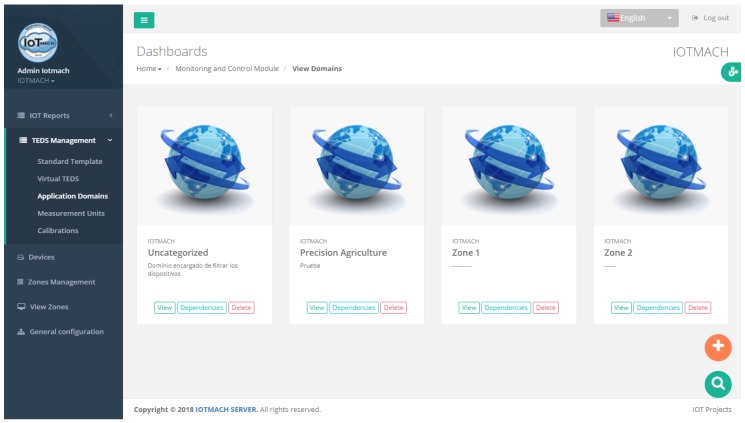
Application domain menu.

**Figure 19:**
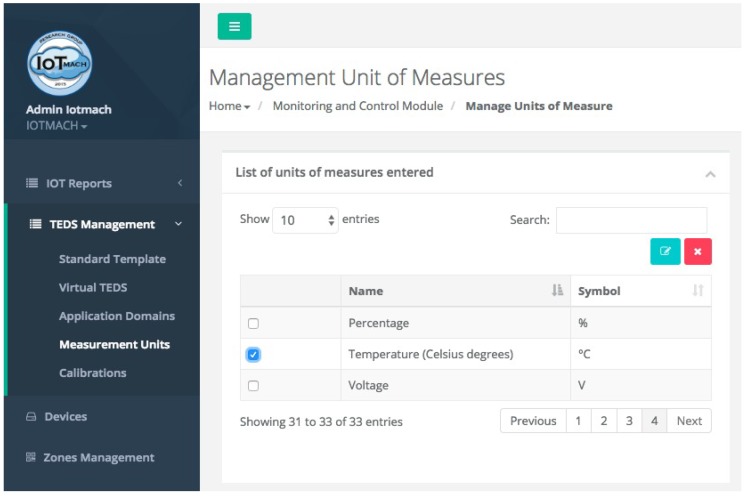
Measurement unit menu.

**Figure 20:**
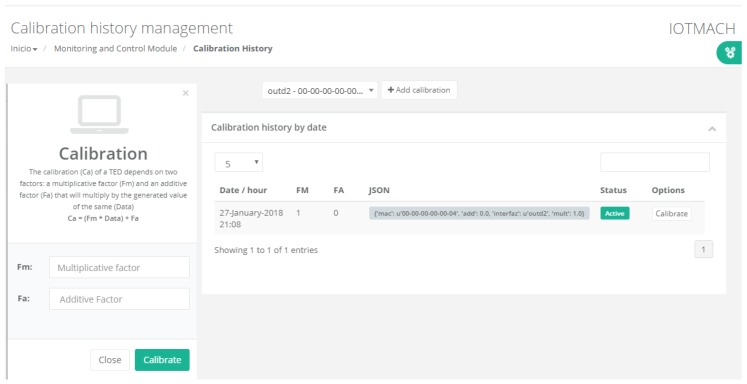
Calibration menu.

**Figure 21:**
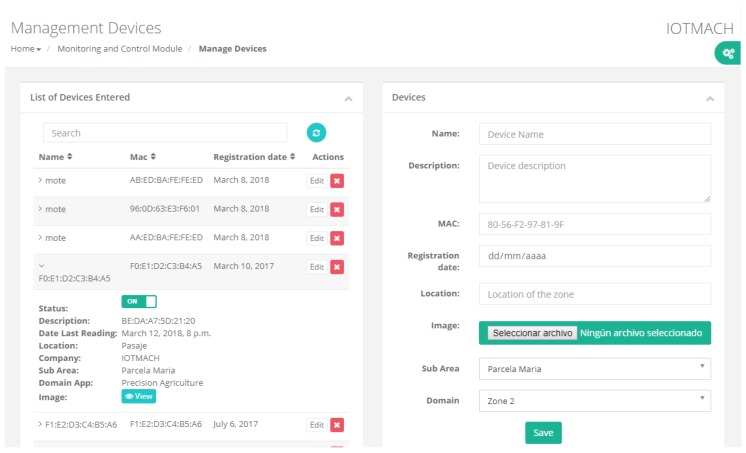
Discovered devices.

**Figure 22:**
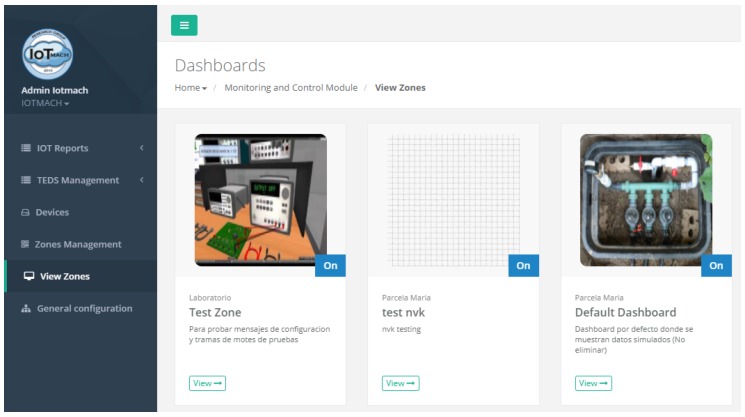
Dashboard list.

**Figure 23:**
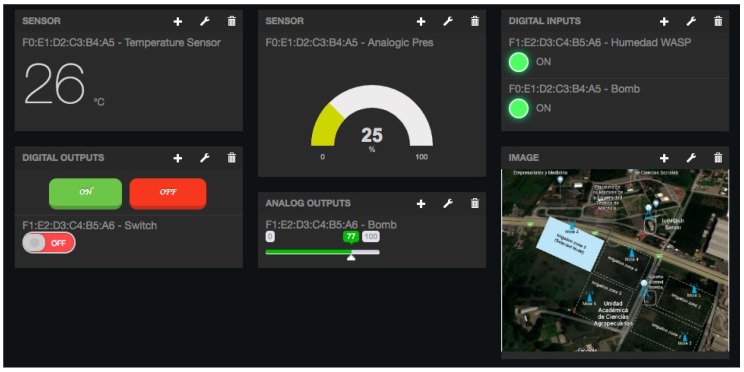
Example of a real-time dashboard.

**Figure 24:**
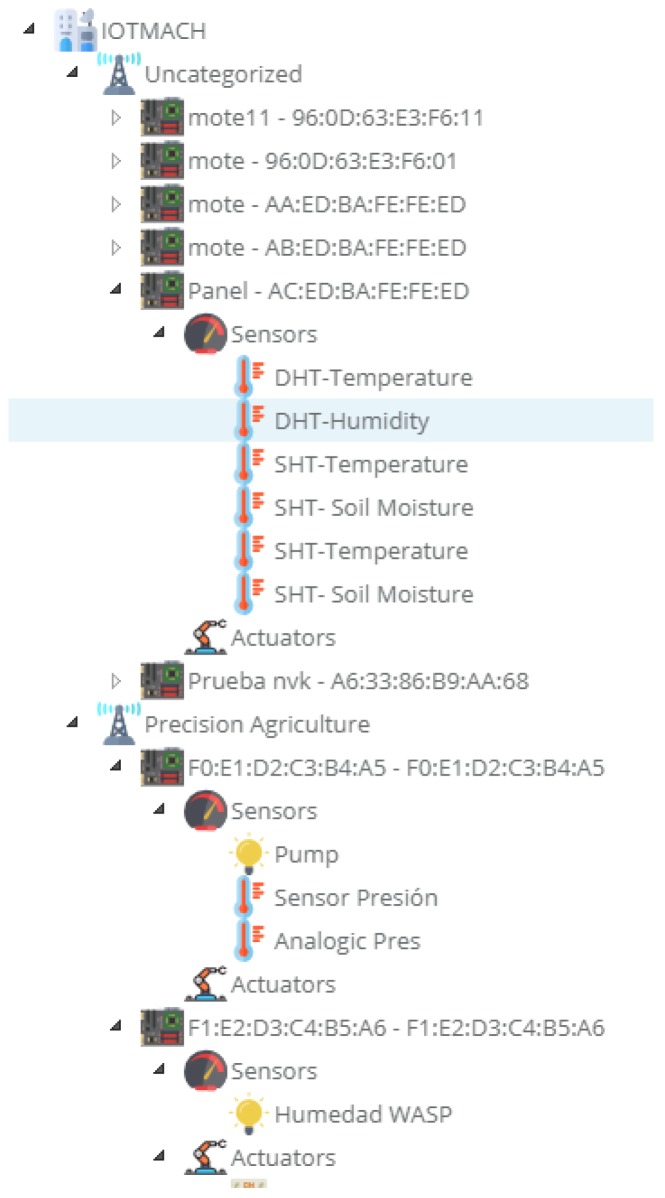
General view tree.

**Figure 25:**
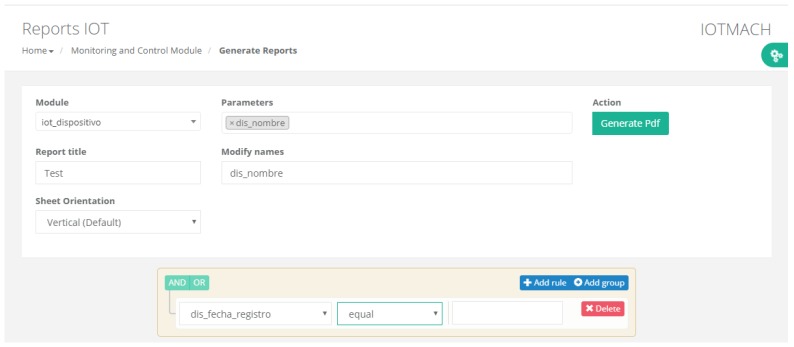
Report configuration.

Figure 15 shows a screenshot of the developed web application. On the left, a list of implemented functions for the management of the necessary TED parameters can be observed:

- Standard template. Figure 16 shows the interface that is used to manage the IEEE 21451 standard templates. Such an interface allows the user to add new templates depending on his/her needs.
- VTEDS. On the left of Figure 17 the extended content of one of the VTEDS listed in Figure 15 is shown. It includes all the descriptors sent by the TIM, so the provided information is not static and can even be edited manually, as can be observed on the right side of the image.
- Application domains. Figure 18 shows the web menu available to create the different application domains manually. The menu also shows the application domains discovered through the TIM configuration frames during the process of self-registration in the cloud.
- Units of measure. Figure 19 shows the menu to manage all types of units of measurement that are necessary for the sensors and actuators available.
- Calibration. The calibration of the sensors and actuators in the architecture occurs dynamically, as explained in Section 3.3.2. Figure 20 shows the interface through which a maintenance technician can add new configuration parameters of one of the discovered sensors. Once the calibration button is clicked, the system sends an MQTT message with the necessary information, which is consumed by the corresponding NCAP. In addition, from this interface, the user can access historical calibrations and send again such previous parameters to a sensor.
- Device management. All the sensor nodes discovered by the cloud, from the moment they perform the auto-registration process, are listed in the menu shown in Figure 21. Once they are detected by the cloud, they can be edited manually.
- Dashboard management. The implemented system is able to generate dashboards dynamically. Thus, the web application allows the user to customize the information to be displayed on the screen, which makes it possible to show together all the available information of different sensors, even the ones of different TIMs. This is performed through the interface shown in Figure 22, which can manage different display areas and choose the sensors and actuators listed in the device management menu. Figure 23 shows an example of a dashboard generated dynamically with real-time information.
- General view. For a better visualization and management of all the elements discovered dynamically in the cloud, there is a structure in the form of drop-down tree (in Figure 24) that allows immediate access to the required information.
- Report management. The system also provides an interface to generate reports from the information stored in the database. Figure 25 shows the interface that allows the user to customize the desired information for the report to be generated.

## 5. Experiments

**Figure 26:**
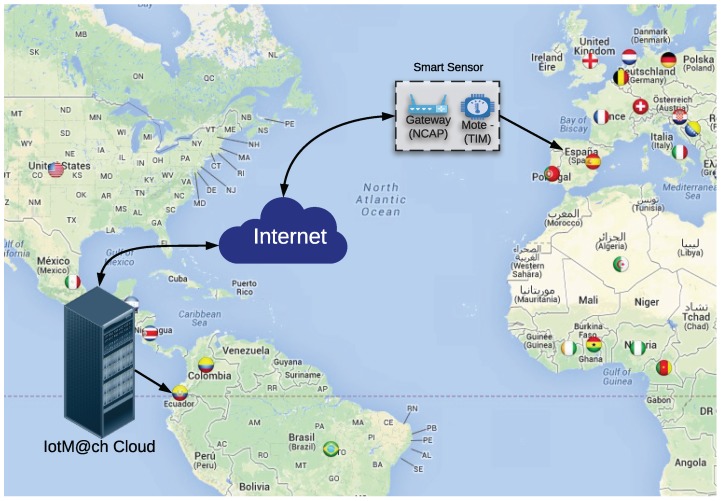
Physical distribution of the IoT platform.

This section shows the evaluation of the proposed architecture in a real IoT environment. In the tests, the cloud was located at the Technical University of Machala (Ecuador) and the sensor nodes (TIM and NCAP) were at the University of Coruña (Spain), as is illustrated in Figure 26.

The proposed experiments were designed to test the system when making use of lightweight protocols and VTEDS. The comparison was carried out by measuring the times required for self-configuration, self-registration, telemetry, and auto-calibration, which allow for evaluating the real impact of the latency in the overall performance of the plug-and-play architecture with three different TIMs: an Eddystone BLE beacon, a BLE GATT-based beacon, and a microcontroller with an Ethernet interface.

In case of the BLE beacons, note that they are small lightweight low-cost low-consumption devices that broadcast certain information packets periodically to indicate its presence or to transmit certain data. For instance, they have been traditionally used to provide location-based services. The BLE Eddystone beacon [83] is based on Eddystone, Google’s open BLE beacon format. The beacon used in the experiments was configured with a beaconing interval of 250 ms, since it was determined in [67] that it offered the best trade-off between latency and consumption when there was Line-of-Sight (LoS) between the beacon and a smartphone, and a 3 m distance between both.

Regarding the BLE GATT-based beacon, it must be noted that the beacon acts like a server that provides services and indicates certain characteristics (the GATT defines the hierarchy and format used to represent and alter BLE data). Thus, to communicate with another BLE device, it is required to first establish a connection (in contrast, Eddystone beacons are connectionless). An in-depth review of the BLE standard is beyond the scope of this paper, but the interested reader can find further details on BLE and GATT in [84].

Finally, with respect to the Ethernet TIM, it must be emphasized that it was selected to determine the architecture’s performance when using a resource-constraint IoT device that uses a wired interface. For the tests performed, the Ethernet TIM consisted basically in an Arduino Mega with an Ethernet shield.

### 5.1. Experimental Setup

**Figure 27:**
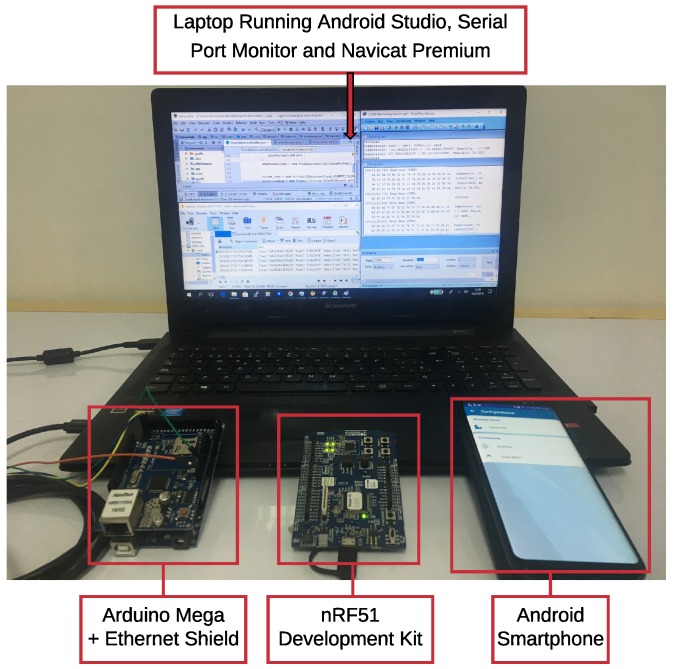
Experimental setup.

Figure 27 shows the elements used in the experiments: a Nordic nRF51 development kit (nRF51-DK) that acts as BLE TIM (both for the Eddystone beacon and for the GATT server), an Android smartphone that acts as an NCAP, and a laptop running Android Studio [85], Serial Port Monitor [86], and Navicat Premium [87]. The main specifications of all these elements are described in Table 2.

In the tests, the speed at which sensor nodes are able to detect and to enable their own hardware (i.e., to perform their self-configuration) was first measured. Next, the time required to detect the sensors when using plug-and-play mechanisms (self-registration), both at the NCAP layer and at the cloud, was determined. After self-registration, both the IoT system and the external users can interact with discovered sensors and actuators. The user experience during such an interaction can be quantified through the telemetry latency, which measures the time needed by the system or a user to receive the sensor data (it starts when the sensor collects a new measurement and stops when the data is available in the NCAP and the cloud). The last measured latency is related to the auto-calibration process, which begins when a user enters new calibration parameters, and it continues until the first calibrated sensor measurement is received. The next subsections describe in detail how such latencies were determined and the obtained results.

### 5.2. Self-Configuration Latency

**Figure 28:**
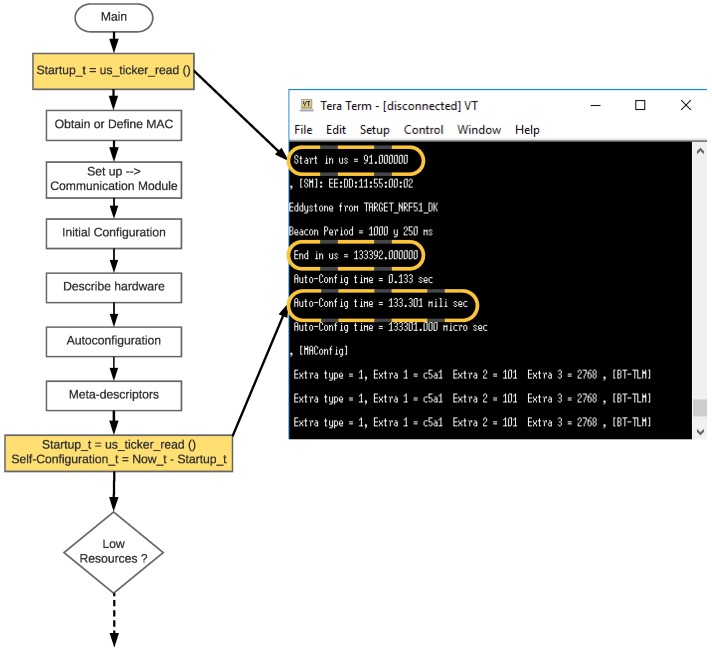
Calculation of the self-configuration latency.

Figure 28 illustrates the measuring procedure, which is based on the flow chart previously presented in Figure 12. The strategy consists in capturing the ticker associated with the beginning and the end of the execution of the functions required by the TIM in order to discover and enable the connected hardware when rebooting the CPU. The difference between such times is the self-configuration latency, whose value can be sent through the serial port and captured by any USB sniffer (e.g., Serial Port Monitor or the Tera Term [88]). In Figure 28, the function *us_ticker_read()* inside the boxes of the flow diagram corresponds to the function used in the online Mbed Integrated Development Environment (IDE) to read the ticker counter of the used CPU (in the performed experiments the firmware of the BLE TIMs was developed with Mbed’s IDE). In Figure 28, it can also be observed a self-configuration latency of 133.301 ms.

Once the TIMs have been programmed, the experiment consisted in turning the TIM off and on several times and then obtaining the averaged latency. The results of the average of 50 tests performed for the three TIMs involved in the self-configuration experiments are 134.268 ms for the Eddystone beacon, 151.53 ms for the GATT server, and 1005.785 ms for the Arduino with the Ethernet shield. It is important to indicate that the measured latency exists only when the TIM firmware is loaded; therefore, in the case of static TIMs or when there are no power cuts, this latency does not impact the system performance. However, as will be observed later in Section 5.6, it greatly influences the total latencies, which worsens the user’s experience when it is required to obtain information from the sensors quickly.

As can be observed, the results obtained for the BLE TIMs are similar, with a difference of only 17.26 ms. In contrast, a lower latency might be expected for the Ethernet TIM due to its wired connection, but the obtained average results were roughly 1 s slower than when using BLE TIMs. Such a difference is explained mainly because of three factors:

- The TIMs use different hardware. Both TIMs use RISC processors that operate at 16 MHz, but the TIM nrf51422 [89] has slightly more resources than the Arduino Mega, since it is based on the ARM Cortex M0 [90] (32-bit CPU and 16 kB RAM), while the ATmega2560 [91] is based on an 8-bit AVR microcontroller with 8 kB SRAM.
- Each TIM makes use of a different development platform. On the one hand, in the case of the BLE TIMs, they were both programmed with Mbed, which is a platform for managing ARM-based IoT nodes that optimizes the firmware for devices with constraint hardware resources. On the other hand, Arduino is a development platform that includes an abstraction layer that allows for simpler and more intuitive programming, even for those who have no experience in IoT, but at the cost of optimizing the managed resources, which impacts configuration latencies and the amount of used program memory.
- The use of the Ethernet shield requires a serial connection with the CPU, which slows the discovery of the Ethernet module.

### 5.3. Self-Registration and Telemetry Latencies

In order to obtain the self-registration and telemetry latencies when using the two proposed BLE TIMs, it was necessary to develop an Android application that acted as an NCAP. In such an application, certain text tags were inserted when an event occurs, so it is possible to use the Android Studio log to determine the timestamps of each event.

**Figure 29:**
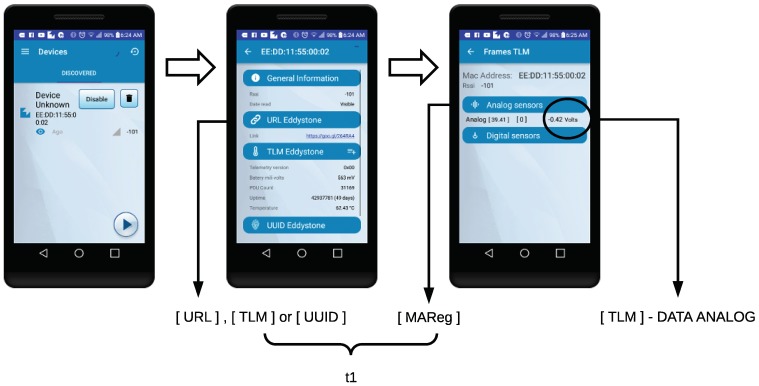
Sequence of screens of the Android application when using the beacon-based TIM.

In the case of the beacon-based TIM, the self-registration latency corresponds to the time elapsed since the NCAP receives the first frame from the Eddystone (either [TLM], [URL] or [UUID]) and until the NCAP receives all the information about the configuration from the TIM (which is marked with the tag [MAReg]). Figure 29 shows the sequence of windows of the Android application that a user goes through when receiving information from a BLE beacon. First, the NCAP discovers a TIM that has entered the area covered by the NCAP’s BLE. The second window is shown when it is received any of the Eddystone frames. The last window is displayed only when the TIM was able to perform the self-registration. In addition, this last window will show dynamically the updated information on the discovered sensors and actuators.

In Figure 29, the self-registration latency is denoted as *t1* and is calculated as indicated in the previous paragraphs, when exchanging the mentioned Eddystone frames. Each time a new sensor arrives, it will be marked with the [TLM]-DATA ANALOG tag, which will be used to obtain the telemetry latency.

**Figure 30:**
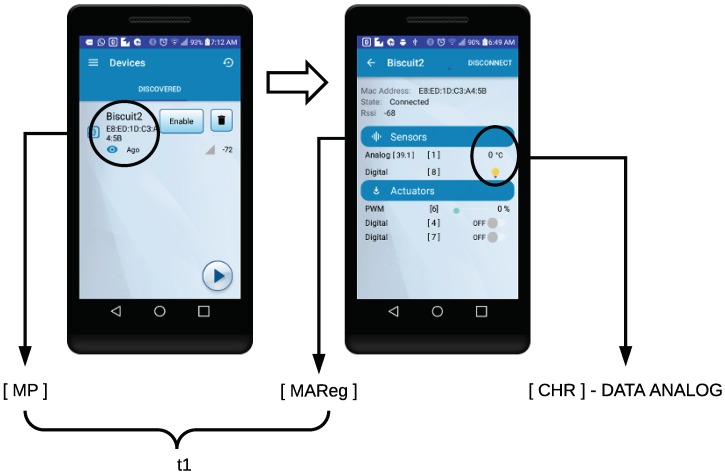
Sequence of screens of the Android application when using the GATT-based TIM.

In the case of the GATT-based TIM, the process to obtain the self-registration latency begins when the user selects the desired TIM from the list of the BLE devices detected by the NCAP. The pairing event of the TIM with the NCAP is marked with the tag [MP], as can be seen in the screenshot on the left of Figure 30. Once the TIM is paired, it sends the configuration of the mote using the LP4S protocol, in order to auto-register in the NCAP (this event uses the [MAReg] label). The screenshot on the right of Figure 30 is generated dynamically based on the TIM hardware discovered (the sensor reading event is marked with the [CHR]-DATA ANALOG tag).

**Figure 31:**
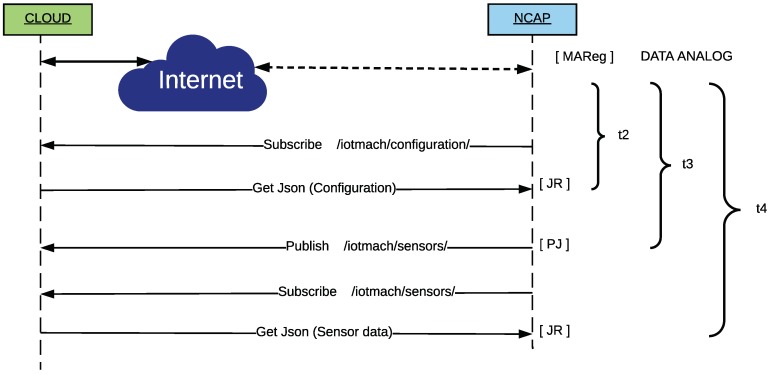
Sequence diagram that illustrates how to obtain the latencies for the BLE TIMs.

Figure 31 shows the sequence of events for both BLE TIMs and the associated tags used to calculate the self-registration latency at the cloud layer (*t2*) and the telemetry latencies at NCAP (*t3*) and cloud (*t4*) layers.

The self-registration latency at the cloud layer (*t2*) represents the time since the first Eddystone frame arrived or the mote was paired, until the configuration frame reaches the cloud. Since the NCAP is connected to the MQTT server of the cloud and subscribed to the configuration topic, it is possible to know when such an information reaches the cloud. During the experiments, this event was marked with the [JR] tag and, although its inclusion involves adding a small delay, it allows for synchronizing the events for their analysis without having to deal with time zone settings due to the location and distance between the cloud and the sensor nodes (remember that the cloud is in Ecuador and the TIM/NCAP are in Spain).

The telemetry latencies for the TIMs are calculated from the time instant when they obtain new data from the sensors (based on a prefixed sampling scheme), until such data reach the NCAP (*t3* in Figure 31). That is, the latency is calculated as the difference between the timestamps associated with the tag [TLM]-DATA ANALOG (Eddystone) or [CHR]-DATA ANALOG, with the tag [PJ], which represents the event that occurs when the NCAP has received all the data from the sensors and then proceeds to publish them in the appropriate MQTT topic of the cloud. Regarding the telemetry latency at the cloud layer (*t4* in Figure 31), it is measured as the difference between the timestamps associated with the DATA ANALOG and the second [JR] tag, which is shown when the published data eventually reach the cloud.

**Figure 32:**
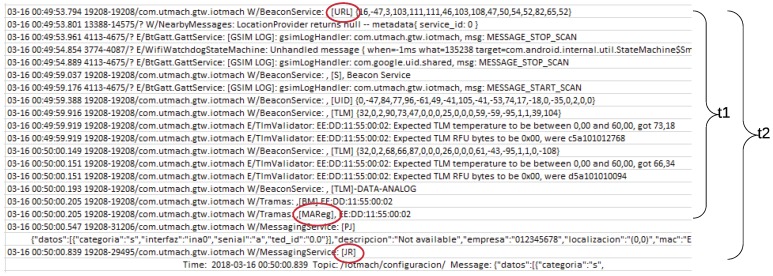
Example of Android log for measuring latencies.

The timestamps in the Android Studio log are calculated in milliseconds for increasing the accuracy during the latency calculations. Figure 32 shows an extract of such a log, where the tags required for the calculation of the self-registration latency in an Eddystone beacon are circled. In this case, the self-registration latency at the NCAP layer (*t1*) was 6.411 s, while the same latency at the cloud layer (*t2*) was 7.04 s.

In the case of the Ethernet TIM, the procedure to obtain the self-registration and telemetry latencies differs with respect to the one used for the BLE TIMs, since the higher layers (NCAP and TIM) vary in hardware/software resources and features. For wired TIMs, it is easy to route the information, so the NCAP can be hosted in an Single-Board Computer (SBC) or inside a virtual machine that runs on a server. In the case of the experiments related to the selected Ethernet TIM, the NCAP was hosted on a CentOS virtual machine and included a local MQTT broker, so the Ethernet TIM published both the configuration and the telemetry frames directly in the NCAP, which sent the information to the cloud.

**Figure 33:**
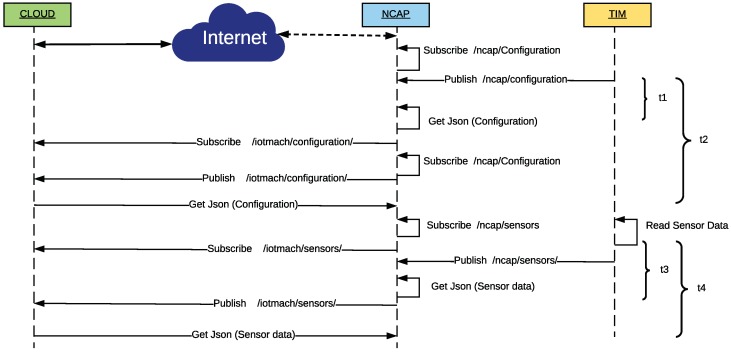
Sequence diagram that illustrates how the latencies for the Ethernet TIM were obtained.

Figure 33 shows the sequence diagram for obtaining both the self-configuration and the telemetry latencies. The self-configuration latency at the NCAP layer (*t1* in Figure 33) is measured from the moment that the TIM recognizes its hardware and publishes it in the topic of configuration of the NCAP, until it extracts the metadata from the received LP4S-JSON. The latency at the cloud layer (*t2*) is measured similarly, but the measurement ends when the NCAP verifies that the JSON has reached the cloud. This is possible since the NCAP also has an MQTT client subscribed to the cloud configuration topic (it is run only for debugging purposes and for obtaining the results of the proposed experiments).

The telemetry latencies at the NCAP (*t3*) and cloud (*t4*) layers are measured from the moment the TIM has a new reading of its sensors until the NCAP extracts the sensor data from the JSON (*t3*) or until such data are made available to the cloud (*t4*).

For all the measurements related to the Ethernet TIM, the timestamps associated with the aforementioned events were extracted from the local database of the NCAP, where each of the JSON frames were published in or read from the configuration or readings topics. To manage the local database and process all the information collected in each experiment, Navicat Premium was used.

The average results obtained from the 45 experiments carried out for each TIM to obtain the self-registration and telemetry latencies of the BLE and Ethernet TIMs are shown in Table 3. In such a table, it can be observed that the self-registration latencies are always greater than those of telemetry, regardless of the hardware and the communication interface. Specifically, there is a difference between the self-registration and telemetry latencies at the NCAP layer of 4.5 s for the Eddystone beacon, 660.5 ms for the GATT server, and 334.67 ms for Ethernet. Similar conclusions can be drawn when comparing the difference between the latencies at the NCAP and the cloud layers: NCAP latencies are always lower, since the NCAP is closer to the TIMs and, in practice, is an intermediary that is necessary for the nodes to reach the cloud.

### 5.4. Auto-Calibration with the Ethernet TIM

**Figure 34:**
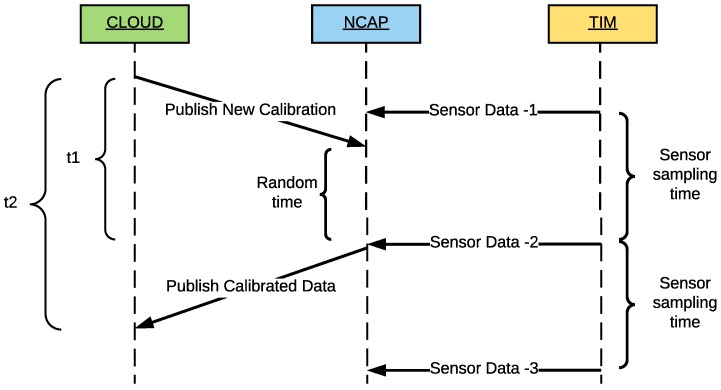
Sequence diagram that illustrates how the auto-calibration latency was calculated.

The auto-calibration latency allows for quantifying the user experience when a technician calibrates one of the self-registered sensors in the IoT system. In a similar way to the previous metrics, the experiments were carried out both at the NCAP and the cloud layers. In both cases, the experiment was performed using the Ethernet TIM, and the associated timestamps were managed from the local database. Both the auto-calibration latency at the NCAP (*t1*) and that at the cloud (*t2*) layers begin to be measured when the user enters new calibration parameters through a web browser. Then, the latency is computed as the time difference between the moment the new calibration parameters are published in the calibration topic and the moment at which the already calibrated sensor data become available in the NCAP (*t1* in Figure 34) or in the cloud (*t2*). Figure 34 illustrates this process. It is worth emphasizing that both measurements are affected by a random time that depends on the reading criterion and the selected sampling time. However, this random time has the same distribution for both latencies without affecting the results.

The results obtained for the 40 auto-calibration tests averaged 3478 ms (*t1*) and 3797.54 ms for *t2*, so it can be observed that there is a difference of 319.54 ms between the NCAP and the cloud layers.

It must be also added that the differences between the NCAP and the cloud experiments are affected by the random latencies of the Internet provider. Although the cloud server has a wired connection to the communications backbone of the university, since the IoT traffic generated by the proposed system does not have priority bandwidth, it competes with the rest of the users of the university campus. Regarding the NCAP, in the experiments, the laptop was connected via WiFi to the Internet like any other regular user of the university.

Comparing the auto-calibration and self-configuration latencies when using the same Ethernet hardware, it can be observed that the difference between both is 2.47 s. Note that the auto-calibration latency is affected by the random time shown in Figure 34, which can vary from 0 s to a maximum of 1 s, which was the sample time indicated for the performed experiments.

### 5.5. Self-Registration and Telemetry Latencies Without Line-of-Sight

**Figure 35:**
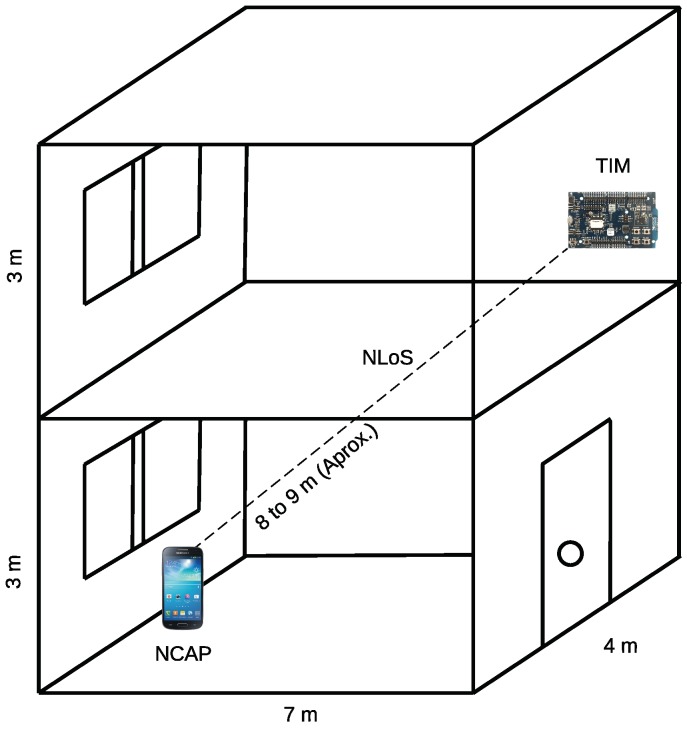
Physical scenario of the experiment with NLoS.

The last experiment was performed in a scenario where there was no Line-of-Sight (NLoS) between the TIM and the NCAP. The objective of the experiment was to determine the impact of the latency in the performance of the infrastructure in real IoT applications where the distance and the presence of obstacles between the NCAP and TIM can compromise the connection and the user experience when managing sensors and actuators. For this experiment, the GATT TIM and a smartphone that acted as an NCAP in the physical scenario depicted in Figure 35 was used. The figure only shows the NLoS scenario, although the same site was used for the LoS scenario but on the same level without obstacles. Note that the NLoS scenario is very aggressive, as it is considered crossing one floor of the building. As can be observed in the figure, both devices are almost 10 m away, which is usually the maximum distance for creating stable BLE connections in most smartphones.

Specifically, the experiments were performed in a building where both the NCAP and the TIM were in small rooms (a bedroom and dining room). The present furniture was mainly made out of plywood, although a few elements contained metal (e.g., there were aluminum chairs and a metallic mattress base). The walls were 9 cm thick and were made out of drywall. The floors were made of light concrete and they were covered with ceramics, having a total thickness of 15 cm. The distance between the transmitter and receiver (NCAP and TIM) never exceeded 10 m, which is usually the maximum supported by the communication standard.

In this case, the experiments were aimed at obtaining the self-configuration and telemetry latencies at both NCAP and cloud layers. The methodology was exactly the same as that described in Section 5.3 for the GATT TIM.

To provide an easy comparison of the metrics mentioned in both scenarios (LoS vs. NLoS), the results obtained for the GATT TIM with line-of-sight are shown in Table 4 together with the ones for the NLoS scenario (in both scenarios the same number of tests were averaged).

The NLoS results show a difference of latencies between the NCAP and cloud layers of 1.36 s for self-configuration and 244.55 ms for telemetry. Comparing the LoS and NLoS scenarios, it was found that the self-registration latency for NLoS at the NCAP was 1.45 s higher than in the LoS scenario, and almost 2 s (1.98 s) at the cloud. In contrast, telemetry latencies at NCAP and cloud layers were similar, being, respectively, 334.14 ms and 344.22 ms greater than in the LoS scenario.

Despite these differences, the results for an NLoS scenario for the GATT BLE TIM are good, considering that a maximum of 4.52 s was required by the self-configuration at the cloud layer and that a minimum of 1.37 s of telemetry latency was obtained at the NCAP layer. These results make the proposed system a valid alternative for implementing BLE-based IoT applications in NLoS scenarios, which are common in home automation applications (due to walls and furniture) or in precision agriculture (because of the foliage and plants).

### 5.6. Analysis of the Results

In the previous section, some initial conclusions were pointed out when analyzing the results of the performed experiments, but it is interesting to go deeper and study the impact of the total latencies from the perspective of the user experience. Specifically, it is worth analyzing the total latencies from the moment a TIM is powered on. Such latencies can be calculated for the LoS or NLoS scenarios using the following expressions:

(1) totalself-registrationtime(atNCAPorCloud)=self-registrationtime+self-configurationtime.

(2) totaltelemetrytime(atNCAPorCloud)=self-telemetrytime+self-configurationtime.

**Figure 36:**
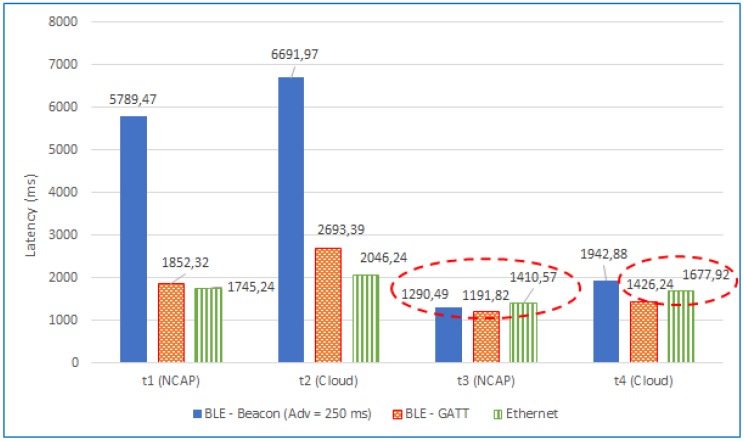
Total latencies for the Line-of-Sight (LoS) scenario.

Figure 36 shows a comparison of such total latencies for an LoS scenario for the three TIMs used in the experiments (*t1* to *t4* are the times previously defined in Figure 33). As can be observed, the total self-registration latencies (*t1* and *t2* calculated by Equation (1)) of the GATT and Ethernet TIMs are similar, the latter being lower in only 107.08 ms at the NCAP layer.

The most interesting findings are related to the telemetry latencies and are highlighted in Figure 36 with red circles. For the total telemetry latency ( *t3* calculated by Equation (2)) at the NCAP layer, both BLE TIMs show the sensor data faster than the Ethernet version (the Eddystone is 120.08 ms faster and the GATT TIM, 218.75 ms quicker). At the cloud layer ( *t4* calculated by Equation (2)), the GATT TIM is 251.68 ms faster than the Ethernet TIM, despite the fact that the experiments were carried out using a remote cloud on the Internet. Therefore, it can be concluded that the fastest telemetry solution corresponds to a BLE GATT TIM, both at NCAP and cloud layers, when the user asks for data from a smartphone in the same place where the sensors are or when it receives them through a remote web browser.

**Figure 37:**
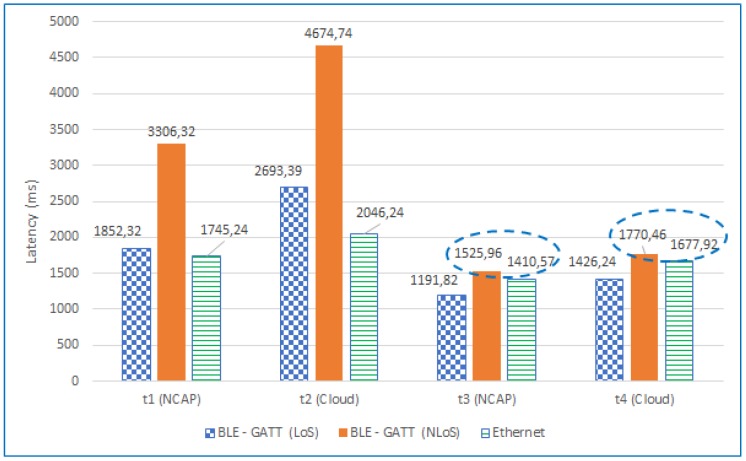
Total latencies for LoS and No Line-of-Sight (NLoS) scenarios.

Finally, Figure 37 compares the total latencies of the GATT TIM in LoS/NLoS scenarios. The total latencies for Ethernet with LoS have been also included.

Once again, the solution with the GATT TIM proves to be a good solution for NLoS IoT scenarios: as can be observed at the view of the values inside the blue circles, there is a difference of only 115.39 ms at the NCAP layer and only 92.54 ms at the cloud layer.

## 6. Conclusions

As a contribution on the evolution of IoT toward WoT, this article presented a generic, flexible, dynamic, and fast plug-and-play architecture based on IEEE 21451 VTEDs, which allow for the interoperability of different types of smart transducers in terms of hardware and communication interfaces. These VTEDS, thanks to the use of lightweight protocols like LP4S, are able to self-describe, self-register, self-configure, and auto-calibrate smart transducers quickly and efficiently.

The article presented the design of the proposed architecture and detailed a web monitoring tool that is able to manage sensors and actuators remotely, to generate self-configuring dynamic dashboards, or to update the calibration parameters of the VTEDS in real time.

In order to determine the performance of the proposed system, it was evaluated in different LoS and NLoS scenarios when making use of three TIMs: two BLE beacons and an Ethernet-based smart sensor. The tests measured diverse latencies with the aim of assessing objectively user experience. The obtained results show that the system is very fast for most application domains and is able to self-register and self-configure in less than 3 s smart sensors placed in Spain, even when the cloud was located in Ecuador. In similar scenarios, the system is able to display sensor data to remote users in less than 2 s, which indicates that a user can access fast the transducer information.

## Figures and Tables

**Table 1:** Examples of Transducer Electronic Data (TED) templates.

Typology	Name	Value/ID
Basic TEDs [30]	Manufacturer ID	43 (Acme Accelerometer Company)
	Model number	7115
	Version letter	B
	Version number	1
	Serial number	X0 1891
Standard templates [29]	Accelerometer & Force	25
	Charge Amplifier (w/attached accelerometer)	26
	Charge Amplifier (w/attached force transducer)	43
	Microphone with built-in pre amplifier	27
	Microphone pre amplifier (w/attached microphone)	28
	Microphones (capacitive)	29
	High-Level Voltage output Sensors	30
	Current Loop Output Sensors	31
	Resistance Sensors	32
	Bridge Sensors	33
	AC Linear/Rotary Variable Differential Transformer (LVDT/RVDT) Sensors	34
	Strain Gauge	35
	Thermocouple	36
	Resistance Temperature Detectors (RTDs)	37
	Thermistor	38
	Potentiometric Voltage Divider	39
Calibration template	Calibration table	40
	Calibration Curve (polynomial)	41
	Frequency Response Table	42
User Data	User name	John Smith
	User ID	123456
	Location	Machala
	Other information	Project 1.2

**Table 2:** Main specifications of the experimental setup elements.

Components	Main Characteristics
Laptop	Lenovo 80E502A5SP G50-80: 15.6${}^{\prime }$${}^{\prime }$, 16 GB RAM, 1 TB HDD
	Intel^®^ Core™i7-5500U CPU @ 2.40 GHz
	OS version: Windows 10-Pro x 64 bits
Android Studio 3.0.1	AI-171.4443003, built on 9 November 2017
	JRE: 1.8.0.152-release-915 amd64
	JVM: OpenJDK 64-Bit Server VM by JetBrains s.r.o
Nordic nRF51-DK	SoC: nRF51822, 2.4 GHz multi-protocol device, 32-bit ARM^®^
	Cortex™M0 CPU with 256 kB/128 kB flash + 32 kB/16 kB RAM
Smartphone	Samsung Galaxy S-8, 4 GB RAM, 64 GB (UFS 2.1) ROM
	Model: SM-G950F
	Android version: 7.0 (Nougat)
	Processor: Exynos 8895, 2.3 GHz Quad + 1.7 GHz Quad, 8 Cores (Octa-Core)
Arduino Mega	SoC: ATmega2560, 8 bits, 16 Mhz
	Digital I/O Pins: 54, Analog Input Pins: 6
	256 kB flash, 8 kB SRAM and 4 kB EEPROM
Ethernet Shield	Ethernet Controller: W5500 with internal 32 K buffer, 10/100 Mb
	connection with Arduino on SPI port.
	Operating voltage 5 V (supplied from the Arduino Board)
Serial Port Monitor	Version 6.0, Build 6.0.235 Eltima Software
	It analyzes serial port activity and monitors several ports within one session.
Navicat Premium	Version 11.0.8, Seamless Data Migration, Diversified Manipulation Tool
	Easy SQL Editing, Intelligent Database Designer, Advanced Secure Connection
	Connect to MySQL, MariaDB, SQL Server, Oracle, PostgreSQL, and SQLite

**Table 3:** Obtained self-registration and telemetry latencies.

TIMs	Self-Registration Latency (ms)		Telemetry Latency (ms)	
	*t1* (NCAP)	*t2* (Cloud)	*t3* (NCAP)	*t4* (Cloud)
BLE Beacon	5655.21	6557.71	1156.23	1808.62
BLE GATT	1700.79	2541.86	1040.29	1274.71
Ethernet	739.46	1040.46	404.79	672.14

**Table 4:** Self-registration and telemetry latencies for the NLoS experiments.

Experiments	Self-Registration Latency (ms)		Telemetry Latency (ms)	
	*t1* (NCAP)	*t2* (Cloud)	*t3* (NCAP)	*t4* (Cloud)
BLE-GATT (LoS)	1700.79	2541.86	1040.29	1274.71
BLE-GATT (NLoS)	3154.79	4523.21	1374.43	1618.93

## Abbreviations

The following abbreviations are used in this manuscript:

AMQP	Advanced Message Queuing Protocol
API	Application Programming Interface
BLE	Bluetooth Low Energy
DIY	Do It Yourself
EEPROM	Electrically Erasable Programmable Read-Only Memory
GATT	Generic Attribute Profile
HAS	Home Automation System
IDE	Integrated Development Environment
IoT	Internet of Things
ISR	Interrupt Service Routine
JMS	Java Messaging Service
LP4S	Lightweight Protocol for Sensors
MQTT	Message Queuing Telemetry Transport
NCAP	Network Capable Application Processor
OGC	Open Geospatial Consortium
QoS	Quality of Service
REST	REpresentational State Transfer
RPC	Remote Procedure Call
SBC	Single-Board Computer
SoC	System-on-a-Chip
SWE	Sensor Web Enablement
TIM	Transducer Interface Module
TED	Transducer Electronic Data Sheet
VTEDS	Virtual TEDS
WoT	Web of Things
WSN	Wireless Sensor Network
XMPP	eXtensible Messaging and Presence Protocol

## References

[1] Leading the IoT Gartner Insights on How to Lead in a Connected World (2017). https://www.gartner.com/imagesrv/books/iot/iotEbook_digital.pdf.

[2] Suárez-Albela M., Fernández-Caramés T.M., Fraga-Lamas P., Castedo L. (2017). A Practical Evaluation of a High-Security Energy-Efficient Gateway for IoT Fog Computing Applications. Sensors.

[3] Robert J., Kubler S., Kolbe N., Cerioni A., Gastaud E., Främling K. (2017). Open IoT Ecosystem for Enhanced Interoperability in Smart Cities—Example of Métropole De Lyon. Sensors.

[4] Fernández-Caramés T. (2015). An Intelligent Power Outlet System for the Smart Home of the Internet of Things. Int. J. Distrib. Sens. Netw..

[5] Blanco-Novoa O., Fernández-Caramés T.M., Fraga-Lamas P., Castedo L. (2017). An Electricity-Price Aware Open-Source Smart Socket for the Internet of Energy. Sensors.

[6] Rodrigues J.J.P.C., De Rezende Segundo D.B., Arantes Junqueira H., Sabino M.H., Prince R., Al-Muhtadi J., De Albuquerque V.H.C. (2018). Enabling Technologies for the Internet of Health Things. IEEE Access.

[7] Fraga-Lamas P., Fernández-Caramés T.M., Castedo L. (2017). Towards the Internet of Smart Trains: A Review on Industrial IoT-Connected Railways. Sensors.

[8] Fraga-Lamas P., Fernández-Caramés T.M. Reverse Engineering the Communications Protocol of an RFID Public Transportation Card. Proceedings of the 2017 IEEE International Conference on RFID (RFID).

[9] Fernández-Caramés T.M., Fraga-Lamas P., Suárez-Albela M., Castedo L. (2017). Reverse Engineering and Security Evaluation of Commercial Tags for RFID-Based IoT Applications. Sensors.

[10] Fraga-Lamas P., Fernández-Caramés T.M., Suárez-Albela M., Castedo L., González-López M. (2016). A Review on Internet of Things for Defense and Public Safety. Sensors.

[11] Pérez-Expósito J.P., Fernández-Caramés T.M., Fraga-Lamas P., Castedo L. (2017). VineSens: An Eco-Smart Decision Support Viticulture System. Sensors.

[12] Fraga-Lamas P., Fernández-Caramés T.M., Noceda-Davila D., Vilar-Montesinos M. RSS stabilization techniques for a real-time passive UHF RFID pipe monitoring system for smart shipyards. Proceedings of the 2017 IEEE International Conference on RFID (RFID).

[13] Fraga-Lamas P., Fernández-Caramés T.M., Noceda-Davila D., Díaz-Bouza M.A., Vilar-Montesinos M., Pena-Agras J.D., Castedo L. Enabling automatic event detection for the pipe workshop of the shipyard 4.0. Proceedings of the 2017 56th FITCE Congress.

[14] Fraga-Lamas P., Noceda-Davila D., Fernández-Caramés T., Díaz-Bouza M., Vilar-Montesinos M. (2016). Smart Pipe System for a Shipyard 4.0. Sensors.

[15] Blanco-Novoa O., Fernández-Caramés T.M., Fraga-Lamas P., Vilar-Montesinos M.A. (2018). A Practical Evaluation of Commercial Industrial Augmented Reality Systems in an Industry 4.0 Shipyard. IEEE Access.

[16] Fernández-Caramés T.M., Fraga-Lamas P., Suárez-Albela M., Vilar-Montesinos M. (2018). A Fog Computing and Cloudlet Based Augmented Reality System for the Industry 4.0 Shipyard. Sensors.

[17] Fernández-Caramés T.M., Fraga-Lamas P., Suárez-Albela M., Díaz-Bouza M.A. (2018). A Fog Computing Based Cyber-Physical System for the Automation of Pipe-Related Tasks in the Industry 4.0 Shipyard. Sensors.

[18] Fraga-Lamas P., Fernández-Caramés T.M., Blanco-Novoa O., Vilar-Montesinos M.A. (2018). A Review on Industrial Augmented Reality Systems for the Industry 4.0 Shipyard. IEEE Access.

[19] Fraga-Lamas P. (2017). Enabling Technologies and Cyber-Physical Systems for Mission-Critical Scenarios. Ph.D. Thesis.

[20] Fernández-Caramés T.M., Fraga-Lamas P. (2018). A Review on the Use of Blockchain for the Internet of Things. IEEE Access.

[21] Fernández-Caramés T.M., Fraga-Lamas P. (2018). A Review on Human-Centered IoT-Connected Smart Labels for the Industry 4.0. IEEE Access.

[22] Patel S.N., Smith J.R. (2017). Powering Pervasive Computing Systems. IEEE Pervasive Comput..

[23] Polianytsia A., Starkova O., Herasymenko K. Survey of hardware IoT platforms. Proceedings of the 2016 Third International Scientific-Practical Conference Problems of Infocommunications Science and Technology.

[24] Kajimoto K., Kovatsch M., Davuluru U. W3C Web of Things. https://w3c.github.io/wot-architecture/.

[25] Khan M., Silva B.N., Han K. (2017). A Web of Things-Based Emerging Sensor Network Architecture for Smart Control Systems. Sensors.

[26] Guinard D., Trifa V. (2016). Using the Web to Build the IoT.

[27] Lee K. IEEE 1451 and IEEE 1588 Standards, 208. https://www.nist.gov/sites/default/files/documents/el/isd/ieee/Information-on-1451_1588-V36.pdf.

[28] Mikhaylov K., Jämsä J., Luimula M., Tervonen J. (2012). Intelligent sensor interface and data format. Intelligent Sensor Networks: The Integration of Sensor Networks, Signal Processing and Machine Learning.

[29] Celicourt P., Piasecki M. (2015). An IEEE 1451.0-based Platform-Independent TEDS Creator using Open Source Software Components. Int. J. Sens. Sens. Netw..

[30] Song E., Lee K. Smart Transducer Web Services Based on the IEEE 1451.0 Standard. Proceedings of the 2007 IEEE Instrumentation Measurement Technology Conference IMTC 2007.

[31] Licht T. (2001). The IEEE 1451.4 proposed standard. IEEE Instrum. Meas. Mag..

[32] Jevtic N., Drndarevic V. (2013). Design and Implementation of Plug-And-Play Analog Resistance Temperature Sensor. Metrol. Meas. Syst..

[33] Higuera J., Hertog W., Perálvarez M., Polo J., Carreras J. (2015). Smart Lighting System ISO/IEC/IEEE 21451 Compatible. IEEE Sens. J..

[34] Kumar A., Srivastava V., Singh M.K., Hancke G.P. (2015). Current Status of the IEEE 1451 Standard-Based Sensor Applications. IEEE Sens. J..

[35] Corotinschi G., Guäitan V.G. The development of IoT applications using old hardware equipment and virtual TEDS. Proceedings of the 2016 International Conference on Development and Application Systems (DAS).

[36] Liu Z., Monte G., Huang V. ISO/IEC/IEEE P21451-001 standard for signal treatment of sensory data. Proceedings of the 2016 IEEE 25th International Symposium on Industrial Electronics (ISIE).

[37] Phala K.S.E., Kumar A., Hancke G.P. (2016). Air Quality Monitoring System Based on ISO/IEC/IEEE 21451 Standards. IEEE Sens. J..

[38] Arduino Official Website. http://www.arduino.cc.

[39] Raspberry Pi Official Website. http://raspberrypi.org.

[40] Ajigboye O.S., Danas K. Towards semantics in wearable sensors: The role of transducers electronic data sheets (TEDS) ontology in sensor networks. Proceedings of the 2016 IEEE 18th International Conference on e-Health Networking, Applications and Services (Healthcom).

[41] Pu F., Wang Z., Du C., Zhang W., Chen N. (2016). Semantic integration of wireless sensor networks into open geospatial consortium sensor observation service to access and share environmental monitoring systems. IET Softw..

[42] Suárez-Albela M., Fraga-Lamas P., Fernández-Caramés T., Dapena A., González-López M. (2016). Home Automation System Based on Intelligent Transducer Enablers. Sensors.

[43] OGC—Sensor Web Enablement (SWE). http://www.opengeospatial.org/ogc/markets-technologies/swe.

[44] Grothe M., Kooijman J., voor Geodesie N.C. (2008). Sensor Web Enablement.

[45] Botts M., Percivall G., Reed C., Davidson J. (2008). OGC^®^ sensor web enablement: Overview and high level architecture. GeoSensor Networks.

[46] Jirka S., Nust D., Schulte J., Houbie F. Integrating the OGC sensor web enablement framework into the OGC catalogue. Proceedings of the 1st International Workshop on Pervasive Web Mapping, Geoprocessing and Services.

[47] Díaz Pardo de Vera D., Sigüenza Izquierdo Á., Bernat Vercher J., Hernández Gómez L.A. (2014). A Ubiquitous sensor network platform for integrating smart devices into the semantic sensor web. Sensors.

[48] Veintimilla-Reyes J., Guillermo J., Vanegas P., Estrella R. SWE Sensor Integration for Controlling Remote Sensors Applied to Hidrometeorological Sensing. Proceedings of the 2017 International Conference on Information Systems and Computer Science (INCISCOS).

[49] Huang C.Y., Liang S.H. (2017). Ahs model: Efficient topological operators for a sensor web publish/subscribe system. ISPRS Int. J. Geo-Inf..

[50] Botts M. (2007). Sensor Model Language (SensorML) | OGC. http://www.opengeospatial.org/standards/sensorml.

[51] O’Reilly T. (2012). OGC^®^ PUCK Protocol Standard | OGC. http://www.opengeospatial.org/standards/puck.

[52] (2012). OGC PUCK Standard Enables ‘Plug and Work’ Sensor Networks | OGC. http://www.opengeospatial.org/pressroom/pressreleases/1542.

[53] Toma D.M., O’Reilly T.C., Bröring A., Dana D.R., Bache F., Headley K.L., Mànuel-Làzaro A., Edgington D.R., Río J.D. (2014). Standards-based plug & work for instruments in ocean observing systems. IEEE J. Ocean. Eng..

[54] Mikhaylov K., Petäjäjärvi J., Mäkeläinen M., Paatelma A., Hänninen T. Extensible modular wireless sensor and actuator network and IoT platform with Plug & Play module connection. Proceedings of the 14th International Conference on Information Processing in Sensor Networks.

[55] Matthys N., Yang F., Daniels W., Michiels T.W.S., Joosen W., Hughes D. μPnP-Mesh: The Plug-and-Play Mesh Network for the Internet of Things. Proceedings of the IEEE World Forum on Internet of Things.

[56] Contiki OS Official Website. http://www.contiki-os.org.

[57] Jabbar S., Ullah F., Khalid S., Khan M., Han K. (2017). Semantic Interoperability in Heterogeneous IoT Infrastructure for Healthcare. Wirel. Commun. Mob. Comput..

[58] RDF 1.1 Primer. https://www.w3.org/TR/rdf11-primer/.

[59] Bröring A., Schmid S., Schindhelm C.K., Khelil A., Kaebisch S., Kramer D., Le Phuoc D., Mitic J., Anicic D., Teniente E. (2017). Enabling IoT Ecosystems through Platform Interoperability. IEEE Softw..

[60] BIG IoT EU H2020 Project-Bridging the Interoperability Gap of the Internet of Things. http://big-iot.eu/.

[61] Gyrard A., Serrano M., Patel P. (2017). Building Interoperable and Cross-Domain Semantic Web of Things Applications. arXiv.

[62] Patel P., Gyrard A., Thakker D., Sheth A., Serrano M. (2016). SWoTSuite: A Development Framework for Prototyping Cross-domain Semantic Web of Things Applications. arXiv.

[63] H2020-ICT-2014-1 European Project FIESTA: Federated Interoperable Semantic IoT Testbeds and Applications. https://cordis.europa.eu/project/rcn/194117_es.html.

[64] OpenIoT—Open Source Cloud Solution for the Internet of Things. http://www.openiot.eu/.

[65] Bröring A., Bache F., Bartoschek T., van Elzakker C.P. (2011). The sid creator: A visual approach for integrating sensors with the sensor web. Advancing Geoinformation Science for a Changing World.

[66] Toma D.M. (2010). Technology Transfer of Observatory Software. https://www3.mbari.org/pw/2010-TTOSInternProject-v1.pdf.

[67] Hernández-Rojas D.L., Fernández-Caramés T.M., Fraga-Lamas P., Escudero C.J. (2018). Design and Practical Evaluation of a Family of Lightweight Protocols for Heterogeneous Sensing through BLE Beacons in IoT Telemetry Applications. Sensors.

[68] MQTT Official Website. http://www.mqtt.org.

[69] Al-Soh M., Zualkernan I. An MQTT-Based Context-Aware Wearable Assessment Platform for Smart Watches. Proceedings of the IEEE 17th International Conference on Advanced Learning Technologies (ICALT).

[70] Ahmed S., Topalov A., Shakev N. A robotized wireless sensor network based on MQTT cloud computing. Proceedings of the 2017 IEEE International Workshop of Electronics, Control, Measurement, Signals and Their Application to Mechatronics (ECMSM).

[71] Sinha A., Sharma S., Goswami P., Verma V.K., Manas M. Design of an energy efficient Iot enabled smart system based on DALI network over MQTT protocol. Proceedings of the 3rd International Conference on Computational Intelligence and Communication Technology (CICT).

[72] Oryema B., Kim H.S., Li W., Park J.T. Design and implementation of an interoperable messaging system for IoT healthcare services. Proceedings of the 14th IEEE Annual Consumer Communications and Networking Conference (CCNC).

[73] Earl B. (2016). Calibrating-Sensors. https://cdn-learn.adafruit.com/downloads/pdf/calibrating-sensors.pdf.

[74] RedBearLab BLE Nano Kit v2—nRF52832. https://www.sparkfun.com/products/14154.

[75] BeagleBone Official Website. http://beagleboard.org/bone.

[76] Orange Pi PC Official Website. http://orangepi.org.

[77] Hernandez-Rojas D., Mazon-Olivo B., Novillo-Vicuña J., Escudero-Cascon C., Pan-Bermudez A., Belduma-Vacacela G. IoT Android Gateway for Monitoring and Control a WSN. Proceedings of the International Conference on Technology Trends.

[78] Amazon AWS Official Website. https://aws.amazon.com.

[79] Microsoft Azure Official Website. https://azure.microsoft.com.

[80] Google Cloud Official Website. https://cloud.google.com.

[81] IOTM@CH—Cloud. http://iotmach.utmachala.edu.ec:8082/iot/conf_Iot.

[82] Google Cloud Platform Pricing Calculator | Google Cloud Platform | Google Cloud. https://cloud.google.com/products/calculator/?hl=es#id=8bcb41f2-8eb1-4baf-8b86-d003aef848a4.

[83] Google Beacon Platform: Eddystone Format. https://developers.google.com/beacons/eddystone.

[84] Davidson R., Townsend K., Wang C., Cufí C. (2014). Getting Started with Bluetooth Low Energy—Tools and Techniques for Low-Power Networking.

[85] Android Studio and SDK Tools | Android Developers. https://developer.android.com/studio/?hl=es-419.

[86] Serial Port Monitor—RS232 Port Sniffer & Analyzer—Serial Monitor. https://www.eltima.com/products/serial-port-monitor/.

[87] Products | Navicat. https://www.navicat.com/en/products.

[88] Tera Term Official Website. https://ttssh2.osdn.jp/index.html.en.

[89] nRF51422 Official Website. https://www.nordicsemi.com/eng/Products/ANT/nRF51422.

[90] Cortex-M0 Official ARM Website. https://developer.arm.com/products/processors/cortex-m/cortex-m0.

[91] ATmega2560 Official Microship’s Website. http://www.microchip.com/wwwproducts/en/ATmega2560.

